# Effective questionnaire-based prediction models for type 2 diabetes across several ethnicities: a model development and validation study

**DOI:** 10.1016/j.eclinm.2023.102235

**Published:** 2023-09-25

**Authors:** Michail Kokkorakis, Pytrik Folkertsma, Sipko van Dam, Nicole Sirotin, Shahrad Taheri, Odette Chagoury, Youssef Idaghdour, Robert H. Henning, José Castela Forte, Christos S. Mantzoros, Dylan H. de Vries, Bruce H.R. Wolffenbuttel

**Affiliations:** a Department of Clinical Pharmacy and Pharmacology, University of Groningen, University Medical Center Groningen, Groningen, Netherlands; b Department of Medicine, Beth Israel Deaconess Medical Center, Harvard Medical School, Boston, MA, USA; c Ancora Health B.V., Groningen, Netherlands; d Department of Endocrinology, University of Groningen, University Medical Center Groningen, Groningen, Netherlands; e Department of Preventive Medicine, Cleveland Clinic Abu Dhabi, Al Maryah Island, Abu Dhabi, United Arab Emirates; f National Obesity Treatment Centre, Qatar Metabolic Institute, Hamad Medical Corporation, Doha, Qatar; g Department of Medicine, Weill Cornell Medicine, Doha, Qatar; h Program in Biology, Division of Science and Mathematics, New York University Abu Dhabi, Abu Dhabi, United Arab Emirates; i Public Health Research Center, New York University Abu Dhabi, Abu Dhabi, United Arab Emirates; j Department of Medicine, Boston VA Healthcare System, Boston, MA, USA

**Keywords:** Prediction, Type 2 diabetes, Machine learning, Prevalence, Incidence, Population screening

## Abstract

**Background:**

Type 2 diabetes disproportionately affects individuals of non-White ethnicity through a complex interaction of multiple factors. Therefore, early disease detection and prediction are essential and require tools that can be deployed on a large scale. We aimed to tackle this problem by developing questionnaire-based prediction models for type 2 diabetes prevalence and incidence for multiple ethnicities.

**Methods:**

In this proof of principle analysis, logistic regression models to predict type 2 diabetes prevalence and incidence, using questionnaire-only variables reflecting health state and lifestyle, were trained on the White population of the UK Biobank (n = 472,696 total, aged 37–73 years, data collected 2006–2010) and validated in five other ethnicities (n = 29,811 total) and externally in Lifelines (n = 168,205 total, aged 0–93 years, collected between 2006 and 2013). In total, 631,748 individuals were included for prevalence prediction and 67,083 individuals for the eight-year incidence prediction. Type 2 diabetes prevalence in the UK Biobank ranged between 6% in the White population to 23.3% in the South Asian population, while in Lifelines, the prevalence was 1.9%. Predictive accuracy was evaluated using the area under the receiver operating characteristic curve (AUC), and a detailed sensitivity analysis was conducted to assess potential clinical utility. We compared the questionnaire-only models to models containing physical measurements and biomarkers as well as to clinical non-laboratory type 2 diabetes risk tools and conducted a reclassification analysis.

**Findings:**

Our algorithms accurately predicted type 2 diabetes prevalence (AUC = 0.901) and eight-year incidence (AUC = 0.873) in the White UK Biobank population. Both models replicated well in the Lifelines external validation, with AUCs of 0.917 and 0.817 for prevalence and incidence, respectively. Both models performed consistently well across different ethnicities, with AUCs of 0.855–0.894 for prevalence and 0.819–0.883 for incidence. These models generally outperformed two clinically validated non-laboratory tools and correctly reclassified >3,000 additional cases. Model performance improved with the addition of blood biomarkers but not with the addition of physical measurements.

**Interpretation:**

Our findings suggest that easy-to-implement, questionnaire-based models could be used to predict prevalent and incident type 2 diabetes with high accuracy across several ethnicities, providing a highly scalable solution for population-wide risk stratification. Future work should determine the effectiveness of these models in identifying undiagnosed type 2 diabetes, validated in cohorts of different populations and ethnic representation.

**Funding:**

10.13039/501100005075University Medical Center Groningen.

**Research in context**

**Evidence before this study**

Type 2 Diabetes (T2D) is an increasingly prevalent condition affecting more than 462 million individuals worldwide. Disease prevention and early detection are crucial to mitigate potentially life-threatening complications as well as healthcare costs. In this setting, using prediction tools is vital to foster population health, mainly through screening. A comprehensive literature search on PubMed (from January 1, 1996, to August 1, 2023) and Medline (from January 1971 to August 1, 2023) showed that there is a knowledge gap concerning T2D prediction models purely based on easy-to-collect questionnaire features. Besides, there is a lack of thorough validation of models trained on White populations among non-White ethnicities. Questionnaire data reflect lifestyle behaviours and health states that play a cardinal role in T2D. It is also evident that certain ethnicities are affected more than others by T2D, facing an earlier onset of the disease and potentially more complications.

**Added value of this study**

This proof of principle study demonstrates that models trained on the White population of the UK Biobank achieved clinically relevant performances for prevalence and incidence prediction across five non-White populations, as well as in the Lifelines external validation cohort. Furthermore, in most instances, these models significantly outperformed the concise Finnish Diabetes Risk Score (FINDRISC) and the Australian Type 2 Diabetes Risk Assessment Tool (AUSDRISK), two widely validated non-laboratory-based clinical risk prediction tools. This demonstrates the potential clinical implications of our models in a wide variety of settings, including non-White populations.

**Implications of all the available evidence**

Deploying these models at a large scale in the primary care setting can be a precise, scalable, and cost-effective means to diagnose positive cases and predict the risk of developing T2D, irrespective of ethnicity. Additionally, resource-limited settings will benefit from using our models by reducing the number of individuals needed to be screened while capturing a significant proportion of the ones developing T2D. To determine the effectiveness of these models in identifying undiagnosed T2D, a follow-up study is required using a cohort where undiagnosed cases can be correctly identified. This effectiveness should be validated in cohorts of different populations and ethnic makeups, as this may vary between these groups.

## Introduction

The number of individuals living with type 2 diabetes mellitus (T2D) is rapidly increasing globally, driven by factors such as ageing, urbanisation, sedentarism, and the increasing prevalence of obesity.^1^, ^2^ In 2019, diabetes accounted for 66.3 million disability-adjusted life years (DALYs) and 4.2 million deaths among adults worldwide,^3^ with disproportionately steep prevalence and complications among non-White ethnic minorities in low-income and middle-income countries.^4^

Populations of non-White ethnic backgrounds are disproportionately affected by diabetes, with a three to five times higher prevalence of T2D than people of White-European background.^5^ South Asians, for instance, usually develop T2D five to ten years earlier and experience a two-to six-fold increased risk of developing T2D compared to White European individuals.^6^ Likewise, 23% of Black African-Caribbean individuals with T2D are diagnosed under the age of 40 years in comparison to only 9% of White Europeans.^7^ Among the predominantly Arab population of the Gulf Cooperation Council countries, T2D prevalence has been suggested to be as high as 25%–36% when undiagnosed case estimates are included and occur at a younger age.^8^ A previous study in the United Arab Emirates showed a prevalence rate of adult T2D and undiagnosed diabetes at 25% and 14.8%, respectively.^9^ Despite the greater incidence and prevalence of T2D and associated comorbidities in these populations, publicly available diabetic registries and validated prediction models for screening or early diagnosis remain scarce.^10^ Existing risk prediction tools in these populations have shown only moderate sensitivity and specificity and are not widely used in clinical practice.^11^ Because of the high rate of undiagnosed diabetics, the prediction of the presence of T2D (prevalence prediction) is essential in the aforementioned settings and highly relevant for lifestyle modification and early treatment initiation to avoid complications and reduced quality of life.

The clinical value of non-laboratory incident T2D prediction tools is well established; however, they lack extensive validation in a wide variety of ethnicities.^12^^,^^13^ Data science, specifically Machine Learning (ML), has shown high potential to further improve risk stratification across a range of clinical applications, including early disease prediction in diabetes.^14^ More importantly, ML-based technologies can accommodate population-wide non-invasive screening, allowing for initial assessments and subsequent referrals.^15^, ^16^, ^17^ Large population cohorts, such as the UK Biobank and Lifelines, constitute a suitable platform for developing and validating data-driven population risk stratification algorithms. These biobanks comprise rich anthropometric, lifestyle, and medical information data, as well as long-term follow-up on disease outcomes of almost 700,000 individuals in total. Of the UK Biobank participants, circa 82% self-identified as “White” and almost 18% self-identified as having a different ethnic background, henceforth referred to as “non-White”, such as “East Asian or South Asian” ancestry, “Black, African, Caribbean, or other Black” ancestries, “Mixed” ancestries, and “Other” ancestries.

In this context, we aimed to develop ML models to predict the prevalence and an eight-year incidence of T2D that could be easily and widely implemented for population screening across multiple ethnicities. In this proof of principle study, we trained questionnaire-based algorithms on the White population of the UK Biobank and validated them internally within the non-White ethnic groups and externally in Lifelines. One challenge with models trained to predict health outcomes is that they can overfit the data they are trained on. This means that the generated models contain an inherent bias toward the training dataset, which can cause the models to perform poorly in practice. Therefore, we validated our models externally using Lifelines to test whether the produced models perform comparably outside the UK Biobank. Finally, we assessed the algorithms’ potential clinical utility against two other ML-based models (containing additional features, i.e., physical measurements and biomarkers) and two gold-standard clinical risk models for the prediction of T2D incidence. Herewith, we showcase significantly enhanced prediction models that can transform primary diabetes care.

## Methods

### Study setting and participants

The UK Biobank is the largest longitudinal population-based cohort, consisting of 502,507 participants aged between 37 and 73 years old, recruited between 2006 and 2010.^18^ For the UK Biobank, ethical procedures are controlled by a dedicated Ethics and Guidance Council (http://www.ukbiobank.ac.uk/ethics). All participants provided written informed consent prior to enrolment. The validation cohort, Lifelines, is a comprehensive and prospective White-European-based population cohort from the northern Netherlands. Lifelines contains data from 168,205 participants aged 0–93 years, with a mean age of 41 years, collected between 2006 and 2013.^19^ Similarly, all participants provided written informed consent prior to enrolment. For a complete overview of the collected data, please see https://biobank.ndph.ox.ac.uk/showcase/catalogs.cgi and https://data-catalogue.lifelines.nl/.

### Type 2 diabetes classification

In the UK Biobank, T2D diagnoses were assigned based on either self-reported T2D, diabetes diagnosed by a doctor, or T2D hospital record annotation based on the International Classification of Diseases (ICD-9 codes 250.X0, 250.X2, and ICD-10 codes E11.X). Supplementary Table S1A demonstrates the data fields associated with the age of diagnosis that were employed to calculate the years until diagnosis from the initial assessment. In cases where more than one age of diagnosis was reported, the lowest reported age was used. We then classified all cases diagnosed before their visit to the assessment centre as prevalent cases, while cases diagnosed after their assessment were annotated as incident cases.

In Lifelines, participants were classified as having prevalent or incident T2D based on self-reported T2D (Supplementary Table S1B). Ages of diagnosis were not asked for during follow-up, and T2D follow-up was only asked for some assessments (2A, 3A, and 3B), while general diabetes follow-up was asked for all assessments (1B, 1C, 2A, 3A, and 3B). Therefore, we estimated the age of T2D diagnosis for every incident case by taking the mean of the age the participant had at the assessment reporting a T2D diagnosis and the age at the previous assessment. To calculate more specific ages of T2D diagnosis, if an incident case had reported a general diabetes follow-up diagnosis before their T2D diagnosis, the mean of the age during that assessment and the previous assessment was used instead to determine the age of T2D diagnosis. According to the National Institute for Health and Care Excellence (NICE) guidelines, the diagnosis of T2D is based on glycated haemoglobin (HbA1c) levels ≥48 mmol/mol, fasting plasma glucose levels ≥7 mmol/L, or random plasma glucose levels ≥11.1 mmol/L.^20^ Unless there are clinical symptoms, these values are not diagnostic of T2D and should be repeated for an individual to be considered as having T2D.^20^ Both in the UK Biobank and Lifelines, the thresholds for “potentially undiagnosed” T2D encompass a plasma glucose level surpassing 7 mmol/L or an HbA1c level exceeding 48 mmol/mol. We set this specific threshold for plasma glucose at 7 mmol/L due to the lack of specification in the UK Biobank records regarding whether glucose readings of individuals were taken while fasting or were random to prevent false negatives in the range of 7.0–11.1 mmol/L.

### Input features

Input features concern the relevant variables used in the modelling procedure of our prediction analyses. Due to the large number of candidate features in the questionnaire, we performed feature selection: we started with an initial list containing all features and sub-selected those with an absolute correlation greater than 0.02 to the target outcome. We then reduced this list to ten features by iteratively extracting the top correlated feature and regressing this feature from the rest of the features. To allow for external validation, we mapped the input features from the UK Biobank to their associated or closest available Lifelines feature (Supplementary Table S2). During feature selection, missing values were imputed using the mean. To investigate whether adding basic measurement and biomarker features improved model performance, we added these features to the questionnaire feature pool and performed feature selection and model training again.

### Data preparation

For the prevalence analyses, everyone with “potentially undiagnosed” T2D was not included in our analysis to avoid bias. This is because, for a T2D diagnosis according to the NICE guidelines, a fasting plasma glucose test above 7 mmol/L, random plasma glucose levels exceeding 11.1 mmol/L, or HbA1c surpassing 48 mmol/mol are not diagnostic of T2D when the individual is asymptomatic and should be repeatedly positive (usually above 7 mmol/L, 11.1 mmol/L, or 48 mmol/mol, at least twice).^20^ The participants of both the UK Biobank and Lifelines that surpass the aforementioned values have not repeated the tests for plasma glucose or HbA1c in a timely manner and, therefore, cannot be considered “undiagnosed cases of T2D”. Besides, in the UK Biobank, individuals have greatly varying fasting times prior to enrolment, conferring uncertainty as to whether individuals with plasma glucose above 7 mmol/L have “potentially undiagnosed” T2D or did not fast long enough. Therefore, to ensure a clean dataset, these cases needed to be excluded from the analysis. For the incidence analyses, we first removed individuals with “potentially undiagnosed” T2D and anyone diagnosed with T2D by a doctor at baseline. Additionally, we removed all incident T2D cases with more than eight years until diagnosis and all persons not developing T2D but not returning to the assessment centre after eight years. Because the different inclusion criteria result in an under-representation of controls, we corrected the incidence in every ethnicity subset by oversampling the controls to obtain the incidence we observed when including remeasured participants only.

### Model training and testing

We set out to predict prevalent and incident T2D across all ethnic groups of the UK Biobank and in Lifelines using questionnaire-based ML models. Self-reported ethnicity was extracted from the UK Biobank, and participants were divided into six different ethnicity groups (Supplementary Table S3). We used Sklearn's LogisticRegression with default settings for model training on the White ethnic population group of the UK Biobank using ten-fold cross-validation.^21^ The model's performance was internally validated in the five other ethnicity categories of the UK Biobank and externally validated in the independent Lifelines cohort. Even though Lifelines is comprised of 98% White individuals, it is imperative to validate our algorithms externally and show that the models can perform independently of the cohort (Supplementary Table S4). Additionally, since our models were trained on the White population of the UK Biobank, the ethnic makeup of Lifelines makes it an appropriate independent cohort for external model validation. All input features were normalised by fitting Sklearn's StandardScaler on the train set and then using this scaler to scale the features in both the train and test sets.

Moreover, we validated the non-laboratory clinical concise Finnish Diabetes Risk Score (FINDRISC) and the clinical Australian Type 2 Diabetes Risk Assessment Tool (AUSDRISK), which employ 9 and 13 features, respectively, spanning medical history, demographics, lifestyle, and anthropometrics, to predict incident T2D.^12^^,^^13^

### Statistical analysis and risk stratification

The predictive performance of the models was assessed through the area under the receiver operating characteristic curve (AUC). AUC values and the associated confidence interval (CI) were calculated using DeLong's method from the R pROC package.^22^ Additionally, AUC values were compared to test for significant differences using the DeLong ROC test from the same package.^22^ To assess the potential clinical utility of the models across different populations, we took a three-step approach to risk stratification. First, we compared the ability of all models to identify individuals at high risk in the general population (including those with and without diabetes for prevalence and those who did and did not develop diabetes for incidence). Youden's method was used to find the risk threshold yielding the best sensitivity/specificity balance. In addition to sensitivity and specificity, Positive Predictive Value (PPV), Negative Predictive Value (NPV), and the respective CI were calculated using the R epiR package.^23^ Then, we simulated another potential application of the incidence models across the different study populations. We stratified the population of every ethnic group into three risk strata based on the individuals' risk of incident T2D (high, medium, and low risk). Each risk stratum contains one-third of the incident T2D cases within each ethnic group. With this analysis, we aim to identify the greatest number of individuals that eventually developed T2D during the follow-up period while minimising the number of people who needed to be screened. Ultimately, to evaluate the improvement in risk prediction provided by our models compared to the abovementioned clinical tools, we conducted a reclassification analysis by calculating the reclassification of events and the categorical Net Reclassification Improvement (NRI) using the R Hmisc package.^24^ Reclassification analysis is a statistical technique that evaluates the effectiveness of a new diagnostic or predictive test compared to an already established one. This method involves classifying people into different risk categories based on the outcomes of both the new and existing tests. The purpose is to determine whether the new test enhances the accuracy of risk categorisation compared to the existing test. The NRI calculates the difference between the proportion of correctly reclassified individuals into higher-risk categories and those who are correctly reclassified into lower-risk categories; higher NRI values indicate that the new diagnostic model is more accurate at correctly predicting outcomes. Specifically, the NRI is the sum of the percentage of reclassified cases and the percentage of reclassified controls. To ensure fair comparisons between models, we matched the sizes of the risk groups in the clinical models with our risk groups, which were determined based on the maximum Youden index.

### Role of the funding source

The funder had no role in study design, data collection, and analysis, decision to publish, or preparation of the manuscript.

## Results

### Baseline characteristics

**Fig. 1:**
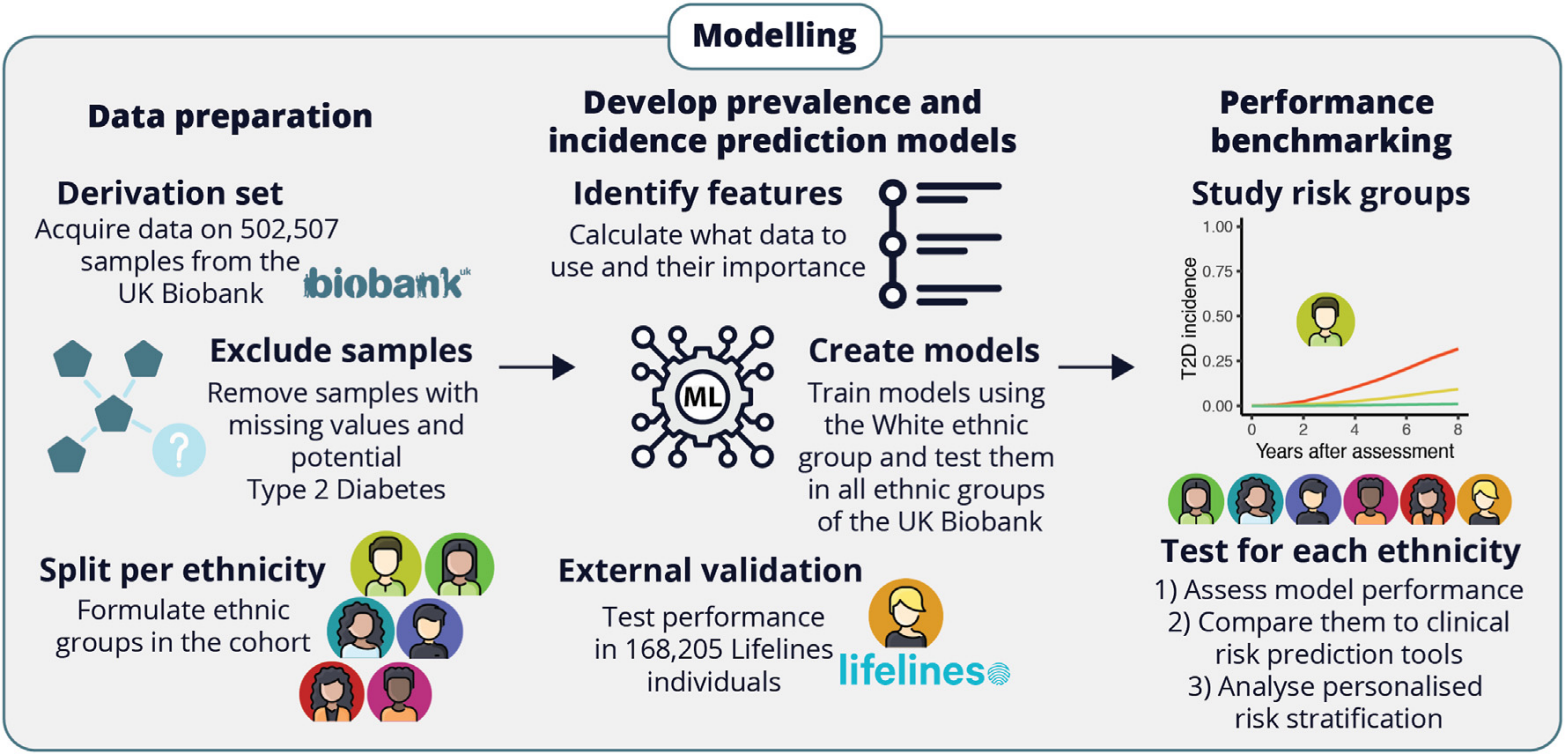
**Workflow showing the steps taken to prepare the data and to create questionnaire-based prediction models for prevalent and incident type 2 diabetes**.

We set out to predict prevalent and incident T2D across all ethnic groups of the UK Biobank and in Lifelines using questionnaire-based ML models (Fig. 1). The included total group size for prevalent and incident T2D prediction models was 631,748 and 67,083 individuals, respectively. Baseline characteristics of the six ethnicity groups and Lifelines are presented in Table 1. Of note, the prevalence and incidence rates of T2D differed greatly between White and non-White populations, with non-White populations having between two-to almost four-fold higher prevalence (12.2–23.3%) and from half to as high as three-fold higher incidence (1.4–8.2%), than the White population of the UK Biobank (6% and 2.8%, respectively). In contrast, Lifelines had a lower prevalence (1.9%) and incidence (1.8%) of T2D compared to White UK Biobank, partly explained by the age differences between these two populations (Table 1).

**Table 1:** Baseline characteristics of the internal and external study populations.

Ethnicities	Internal training cohort	Internal testing cohort					External validation cohort
	White (n = 472,696)	South Asian (n = 8,024)	Caribbean (n = 5,137)	East Asian (n = 4,263)	Black (n = 3,969)	Other (n = 8,418)	Lifelines (n = 168,205)
**Type 2 Diabetes**							
Prevalence	6%	23.3%	15.6%	13%	15.9%	12.2%	1.9%
Incidence	2.8%	8.2%	5.6%	6%	1.4%	3.2%	1.8%
**Questionnaire features**							
Male	45.7%	53.8%	36.9%	45.8%	48.6%	45.7%	42.2%
Female	54.3%	46.2%	63.1%	54.2%	51.4%	54.3%	57.8%
Age	58 (13)	53 (14)	51 (12)	52 (14)	50 (13)	53 (14)	43 (18)
Aspirin	14%	18.5%	12.5%	11.6%	13.8%	13.8%	99.7%
Blood pressure medication	20.6%	27.1%	28.4%	19.3%	30.6%	20.6%	8.4%
Body mass index (BMI)	27.4 (4.8)	27.3 (4.5)	29.2 (5.6)	25.8 (4.3)	29.5 (5.2)	27.8 (5.1)	25.6 (4.6)
Bread intake (slices/week)	10 (10)	8 (8)	6 (6)	8 (10)	8 (10)	10 (9)	22.5 (19.5)
Coronary artery disease before the first assessment	3.3%	5.8%	1.6%	3.2%	1.6%	3.1%	1.5%
Cholesterol lowering medication	17.3%	26.5%	15.1%	18.5%	15.7%	17.6%	4.9%
Dentures	16.9%	9.7%	20.1%	15.5%	10%	14.4%	8.5%
Siblings history of diabetes	7.8%	25.9%	18.6%	18.4%	12.7%	14.6%	1.3%
Parents history of diabetes	16.5%	43.3%	45.8%	34.6%	23.5%	27.2%	6.4%
Number of medications taken	2 (4)	2 (4)	2 (4)	1 (3)	2 (4)	2 (4)	1 (2)
Pack years of smoking (0 years not included)	19.5 (22.5)	15 (15.5)	13.2 (14.2)	15 (16.6)	13.8 (15.1)	16.8 (19.3)	8.5 (13)
Slow walking pace	7.8%	17.7%	12.2%	15.4%	15.6%	15%	N/A
Time spent watching television (TV) (hours/day)	2.8 (1.7)	2.5 (1.7)	3.3 (2.2)	2.4 (1.8)	2.7 (2.1)	2.5 (1.9)	2.3 (1.6)
Unable to work because of sickness or disability	3.9%	7%	7.1%	4%	5.7%	6.9%	2.9%
Gained weight in the past year	27.8%	29.6%	39.7%	28%	36.1%	30.9%	20.9%
Lost weight in the past year	15%	15.3%	19.3%	14%	18%	17%	20.9%
Alcohol intake frequency							
Daily or almost daily	21%	6.6%	9.2%	8.8%	4.9%	10.7%	10.9%
Three or four times a week	23.8%	8.1%	12.3%	9.8%	7.3%	12.6%	10.3%
Once or twice a week	26.3%	13.2%	23.3%	17%	16%	18.6%	39%
One to three times a month	11.2%	7.2%	15.8%	10.3%	10.5%	11%	19.5%
Special occasions only	10.9%	16.7%	26.1%	26.9%	28.9%	22%	N/A
Never	6.8%	47.6%	13.1%	27.1%	31.9%	23%	20.3%
Glucosamine intake	19.3%	11.6%	14.7%	17%	11.1%	14.1%	N/A
**Basic measurements**							
Seated height (cm)	138 (9)	133 (13)	135 (13)	134 (13)	134 (13)	136 (13)	N/A
Waist circumference (cm)	90 (19)	92 (15)	91 (17)	86 (17)	93 (16)	90 (18)	89 (17)
Mean heart rate (bpm)	58 (10)	59 (9)	60 (8)	58 (9)	60 (9)	59 (9)	72 (11)
Mean diastolic blood pressure (mmHg)	81 (14)	82 (14)	84 (14)	81 (14)	84 (14)	81 (14)	72 (13)
**Biomarker features**							
Total cholesterol (mmol/L)	5.71 (1.14)	5.29 (1.12)	5.34 (1.09)	5.52 (1.12)	5.18 (1.1)	5.51 (1.15)	5.01 (1.02)
Gamma glutamyltransferase (U/L)	37.3 (42.2)	36.7 (39.2)	40.4 (40.3)	34.8 (35.3)	42.8 (42.4)	38.5 (43.3)	26.3 (25.7)
Glucose (mmol/L)	5.1 (1.2)	5.4 (1.9)	5.1 (1.5)	5.2 (1.5)	5.1 (1.5)	5.3 (1.7)	5 (0.8)
Glycated haemoglobin (HbA1c) (mmol/mol)	36 (6.5)	40.8 (10.6)	39.1 (9.5)	38.3 (8.4)	38.9 (10.1)	38 (9.3)	37.1 (4.9)
High light scatter reticulocyte (%)	0.4 (0.3)	0.4 (0.2)	0.5 (0.2)	0.4 (0.2)	0.5 (0.3)	0.4 (0.3)	N/A
Neutrophil count (10^9^ cells/L)	4 (1.7)	4.2 (1.7)	3.1 (1.8)	3.9 (1.7)	2.8 (1.4)	3.9 (1.8)	3.1 (1.4)

Data are presented as the mean (standard deviation) unless otherwise noted. The basic measurements are presented as median (interquartile range).

### Contribution of questionnaire features

**Fig. 2:**
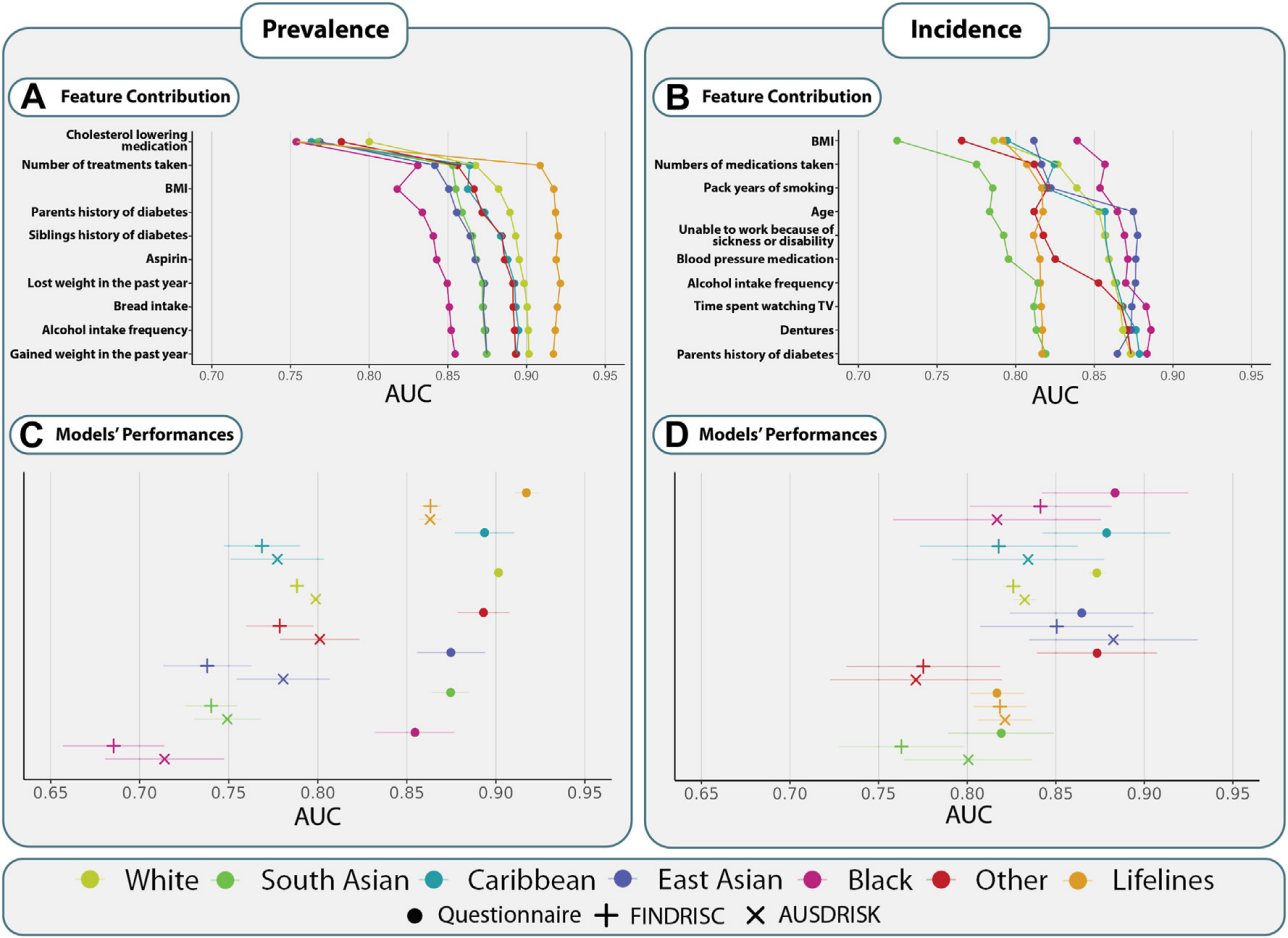
**Feature contribution and performance of type 2 diabetes prediction models for prevalence and incidence.** A list of predicting features included in our models for prevalence **(A)** and incidence **(B)** prediction and their contribution to the models' performance is presented. Below, the performance of different models across populations for prevalence **(C)** and incidence **(D)** is shown. Each colour-symbol combination refers to a specific model and population, explained in detail in the bottom panel. The AUC and 95% Cl are shown for all models. BMI, body mass index; AUC, area under the receiver operating characteristics; TV, television; FINDRISC, Finnish Diabetes Risk Score; AUSDRISK, Australian type 2 diabetes risk assessment tool; T2D, type 2 diabetes; CI, confidence interval.

The correlation between different questionnaire features pertaining to nutrition, smoking, physical activity, medication, and medical history and prevalent or incident T2D for each population are presented in detail in Supplementary Fig. S2A and B. The contribution of each feature to the prevalence and incidence model is shown in Fig. 2A and B. Both prevalence and incidence models put high importance on Body Mass Index (BMI) and the number of medications taken, positioning them in the top three features of both models. Furthermore, incidence includes a feature representing sedentarism (time spent watching television (TV)). We observe an evident performance saturation with five to six input variables, particularly for prevalence prediction.

### Performance of type 2 diabetes prediction models

With ten questionnaire features, the performance of prevalence prediction models measured by their AUC ranged from 0.855 to 0.901 (Fig. 2C and Supplementary Fig. S3A) within the UK Biobank populations and an AUC of 0.917 in the independent validation cohort Lifelines. For models predicting incident T2D in the UK Biobank, AUCs ranged from 0.819 to 0.883 (Fig. 2D and Supplementary Fig. S3B), while in Lifelines, the AUC was 0.817. The detailed performance metrics of the questionnaire-only models are shown in Supplementary Tables S5A and B.

Additionally, we performed an exploratory analysis of the potential added benefit of two other types of models: one including basic physical measurements and one including blood biomarkers (Supplementary Figs. S4A, S4B, S5A, S5B, S7A, S7B, S8A, and S8B). For prevalence prediction, including basic measurements significantly improved the performance of questionnaire-only models for all UK Biobank populations, except for Other, yet lowered the AUC of Lifelines (Supplementary Table S8A, Supplementary Fig. S10). In contrast, for incidence prediction, adding basic measurements significantly increased the performance of only two populations, UK Biobank White and Lifelines, though all populations showed higher AUCs. Including biomarkers led to a significant improvement in all instances except for incidence prediction among the Black population, where the Questionnaire-only models seem to yield a marginally higher performance (Supplementary Fig. S10 and Supplementary Tables S8A, S8B). The feature importance of these models is shown in Supplementary Figs. S4A, S4B, S7A, and S7B.

### Comparison with non-laboratory clinical risk models

We also compared the questionnaire-only models to two clinically validated non-laboratory risk scores. First, we tested the performance of the concise FINDRISC, developed as a simple screening tool for individuals at high risk of developing T2D. We observed that the questionnaire-based models significantly outperformed FINDRISC for prevalence prediction in all populations, and they significantly outperformed FINDRISC in four out of seven populations for predicting incidence (Fig. 2C and D, and Supplementary Tables S9A, S9B). Similarly, the questionnaire-based models significantly outperformed the AUSDRISK models in all prevalence predictions as well as in three out of seven populations for incidence prediction (Fig. 2C and D, and Supplementary Tables S9A, S9B). In all other instances, there were no significant differences; however, our models yielded overall higher AUCs.

### Sensitivity analysis and clinical utility of risk stratification

Furthermore, we conducted an in-depth sensitivity analysis of the risk stratification for all models to assess their potential clinical utility (Supplementary Tables S5A, S5B, S6A, S6B, S7A, and S7B). Based on the thresholds provided by the Youden index, the questionnaire-only models obtained very high sensitivity-specificity balance, PPV, and NPV. Both sensitivity and specificity were consistently high (above 74% and 83% for prevalence and 75% and 68% for incidence, respectively) for all populations. The corresponding NPVs for all models were above 93% and 98% for prevalence and incidence, respectively. For the models including biomarkers, further improvement in the sensitivity-specificity balance was seen, with a lower proportion of individuals identified as high risk also translating to higher PPV across the populations for prevalence and incidence. All corresponding NPVs were above 97% and 99% for prevalence and incidence, respectively.

**Fig. 3:**
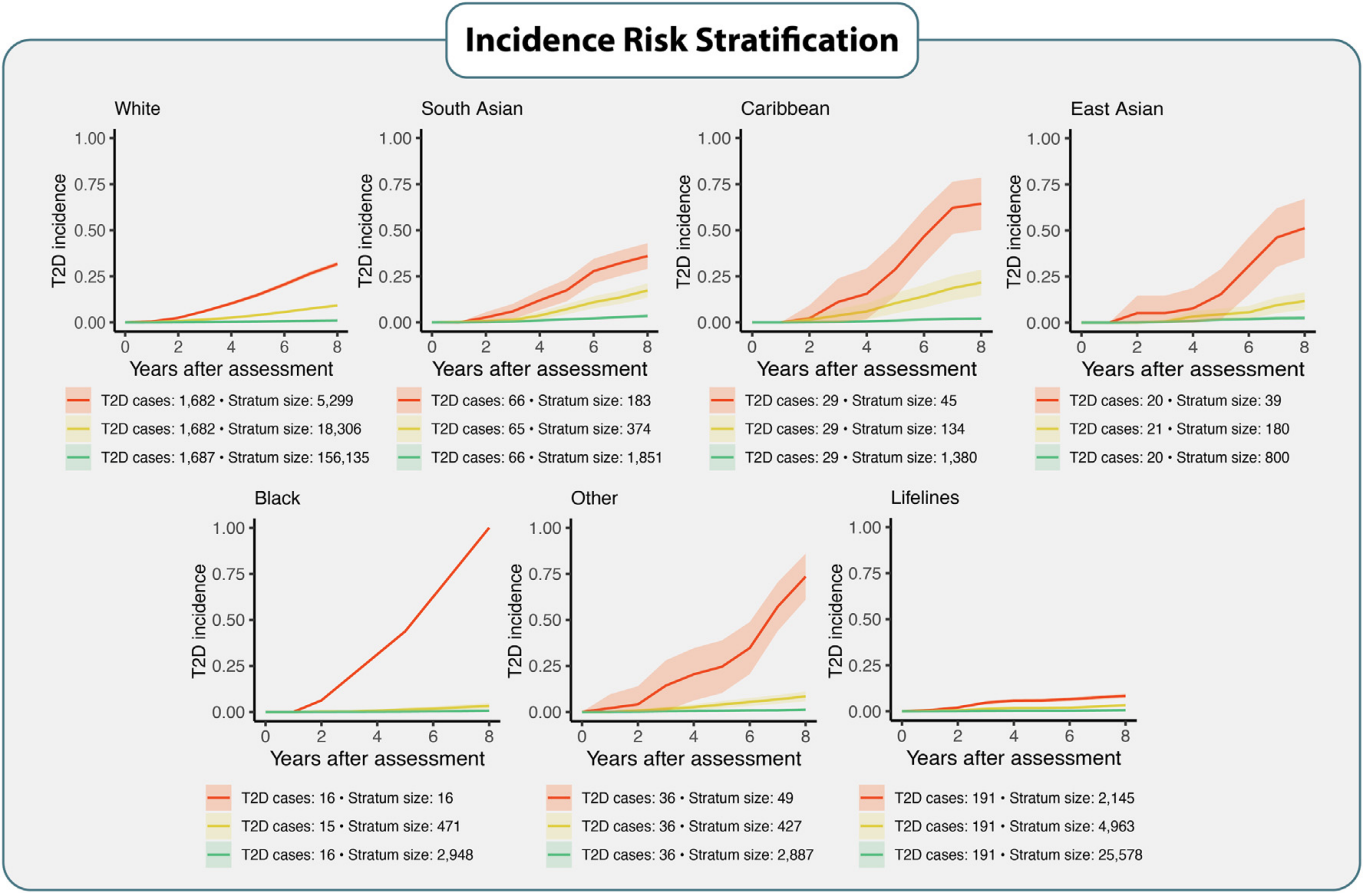
**Risk identification for developing T2D per population**. Every population is separated into three risk strata, according to the individuals' risk of incident T2D (high risk = red, medium risk = yellow, low risk = green), with each risk stratum containing 33% of all T2D cases. The x-axis represents the interval of years between the biobank entry and the moment of receiving a diagnosis of T2D. The y-axis represents the incidence of T2D. The stronger-coloured lines represent the average T2D incidence within each risk stratum, and the lighter-coloured bands around the lines show the 95% CI. T2D cases correspond to the total number of T2D incident cases within each risk stratum. Stratum size corresponds to the number of individuals within each risk stratum. Stratum sizes show how many individuals must be screened to identify 33% of all T2D cases within each risk-stratum. T2D, type 2 diabetes; CI, confidence interval.

In the second step of the analysis, we separated each population into three risk strata (∼33% of the T2D incident cases in each risk stratum) based on the individuals’ risk of T2D eight-year incidence. We observed that the questionnaire-only models could identify small groups of very high-risk individuals who eventually developed T2D during the follow-up period (Fig. 3). By screening as little as 0.47% (Black population) to 7.6% (South Asian) of individuals from different ethnic populations (belonging to the high-risk strata), the questionnaire-only models identified 33% of individuals who developed T2D within each ethnic group. In the high-risk strata, the average incidence of T2D was at least ten-fold higher compared to the lowest-risk strata (Fig. 3). The models also identify 66% of all individuals who developed T2D (belonging to the high- and medium-risk strata) while screening only between 11.5% (Caribbean population) to 23.1% (South Asian population) of all individuals across different ethnic populations. These slightly larger high- and medium-risk strata also show at least a six-fold higher risk across all populations compared to lowest-risk population strata. For the two other types of models (with additional physical measurements and the ones with the addition of biomarkers), the highest-risk strata generally showed even higher average incidence despite the similar size (Supplementary Figs. S6 and S9). For all ethnicities, 66% of incident T2D cases (including high-risk and medium-risk individuals) could be identified by screening less than 10% of each ethnic population using the model, including biomarkers.

### Reclassification analysis

Ultimately, the reclassification analysis demonstrates that in almost all cases, our models correctly reclassify more cases than the clinically established prediction tools FINDRISC and AUSDRISK (Table 2). Notably, for the White, Caribbean, Other, and South Asian populations, our models correctly reclassify more events reaching statistical significance compared to FINDRISC (Table 2). Compared to AUSDRISK, our models reach statistical significance among the White and Other populations in correctly reclassifying T2D cases, along with statistically significant NRI values (Table 2, Supplementary Table S10A). The addition of physical measurements overall reclassifies more events correctly and seems to perform better in Lifelines compared to the Questionnaire Models (Supplementary Table S10B). The models also including biomarkers, largely outperform the clinical tools by reaching statistical significance in almost all instances (Supplementary Table S10C). The high/low-risk group reclassifications, along with NRIs and reclassification of non-event percentages, are demonstrated in detail in Supplementary Tables 10A–C.

**Table 2:** Reclassification analysis comparing our questionnaire-based models to FINDRISC and AUSDRISK for incidence prediction.

Risk model	Population	Reclassification events %	Reclassification events N per 10,000	P-value
FINDRISC	White	6.4 (5.2–7.6)	637 (519–756)	<0.001
FINDRISC	Black	2.2 (−5.2 to 9.5)	217 (−518 to 953)	0.6
FINDRISC	Caribbean	12.6 (3.7–21.5)	1264 (374–2154)	0.005
FINDRISC	East Asian	9.8 (−2.8 to 22.4)	984 (−278 to 2245)	0.1
FINDRISC	Other	14.8 (6.4–23.3)	1481 (637–2326)	<0.001
FINDRISC	South Asian	12.7 (6.1–19.3)	1269 (610–1928)	<0.001
FINDRISC	Lifelines	−2.8 (−6.3 to 0.7)	−279 (−627 to 69)	0.1
AUSDRISK	White	5.9 (4.4–7.4)	591 (441–741)	<0.001
AUSDRISK	Black	3.4 (−8.2 to 15.1)	345 (−819 to 1509)	0.6
AUSDRISK	Caribbean	5.7 (−3.9 to 15.3)	571 (−389 to 1532)	0.2
AUSDRISK	East Asian	0 (−16.6 to 16.6)	0 (−1656 to 1656)	1
AUSDRISK	Other	25.6 (14.7–36.6)	2564 (1472–3656)	<0.001
AUSDRISK	South Asian	7.8 (−0.9 to 16.4)	776 (−91 to 1642)	0.08
AUSDRISK	Lifelines	0.4 (−3.7 to 4.4)	38 (−365 to 441)	0.9

Reclassification events % correspond to our models' net percentage of reclassified individuals with T2D compared to the clinically established tools. Reclassification of events per 10,000 events corresponds to the net number of T2D cases reclassified when screening 10,000 cases. Positive reclassification events indicate that our models correctly reclassify more cases than the other two clinical tools, whereas negative events indicate the opposite. The reclassification events percentages (%) and reclassification events N per 10,000 are presented along with the 95% CI. FINDRISC, Finnish Diabetes Risk Score; AUSDRISK, Australian type 2 diabetes risk assessment tool; T2D, type 2 diabetes; CI, confidence interval.

## Discussion

In this study of over 600,000 individuals for prevalence and over 67,000 for incidence prediction, we showed for the first time that questionnaire-based ML models can accurately predict T2D prevalence and eight-year incidence across all ethnicities present within the UK Biobank, as well as the Lifelines external validation cohort. For almost all ethnicities, these models outperformed two established clinically validated T2D risk assessment tools. Despite the performance improvement verified with the addition of blood biomarkers, the questionnaire-only models showed clinical utility for detecting prevalent and incident T2D.

Previous research on the performance of prediction models for incident T2D has shown substantial differences across ethnicities. A re-estimation of the Atherosclerosis Risk in Communities (ARIC) model for the prediction of five-year diabetes risk in the Coronary Artery Risk Development Study in Young Adults (CARDIA) cohort showed significant differences in performance between White and African Americans (AUC 0.902 vs 0.816).^25^ Another study of 12,043 Black and White individuals focusing on T2D prediction using anthropometric features and lipid levels reported an AUC of 0.79.^26^ In this study, we observed less variation in the model performances between White and Black individuals for both prevalent and incident T2D prediction. The models developed herein overall outperform what has been previously demonstrated in Black populations, even without glucose as an input feature, and contradict the results of previous analyses that suggested that risk scores trained on the European-descent population are not applicable to other ethnic groups.^26^^,^^27^ Additionally, our questionnaire-based models significantly outperformed FINDRISC and AUSDRISK across all seven populations for prevalent T2D detection. For incidence, our models outperformed the above-mentioned tools in four populations compared to FINDRISC and three populations compared to AUSDRISK. This is especially relevant since both FINDRISC and AUSDRISK have been shown to perform only moderately well in several non-White populations,^28^^,^^29^ despite AUSDRISK including ethnicity as an input feature and being intended to be used in the ethnically diverse Australian population.^30^ As expected, adding blood biomarkers to the models resulted in further improvements in predictive performance with AUCs generally above 0.90, mainly due to high correlations conferred by these features (Supplementary Figs. S7A, S7B, S10). Despite being significant, these improvements in AUC were not substantial enough to unequivocally justify their deployment over the questionnaire-only models considering the practical challenges discussed further in detail below.

As such, the goal of population-level risk stratification is not merely to predict individual risk accurately but to clearly distinguish groups with different levels of risk.^31^ To assess the potential stratification utility of our models, we first optimised their sensitivity-specificity balance with the Youden index. We found that all models achieved high to very high sensitivity and specificity for both prevalence and incidence prediction across all ethnicities. Given the low prevalence and incidence of T2D in White populations, a high specificity and NPV were expected for the White UK Biobank population and Lifelines. However, specificity and NPV remained high even in other ethnicities with higher prevalence and incidence rates (Supplementary Tables S5A, S5B, S6A, S6B, S7A, and S7B). The main difference with the addition of biomarkers was the increase in PPV, stemming from the lower number of individuals identified as high risk (between 20% and 29% for questionnaire-only predictions and generally around 18% when biomarkers were included). However, we also aimed to assess the usefulness of the models in settings where resources are limited, or population health data is lacking, and where it is essential to accurately identify as many high-risk individuals as possible while minimising the number of screened individuals. In such instances, screening more than a quarter of the population might be prohibitive from a cost and logistics perspective, hampering the model's clinical utility. Herein, we demonstrated that all models can also be applied to identify smaller groups of individuals at very high risk and that 33% and 66% of all incident diabetes cases can be identified by screening less than 10% and 23% of the population using the questionnaire-only models, respectively. Additionally, by demonstrating high predictive abilities for T2D prevalence, our models will be valuable for early diagnosis, especially in areas where T2D is underdiagnosed and often missed. This is essential for minimising complications and decreased quality of life associated with late T2D diagnosis.

The data from these two simulated scenarios suggests that while there is a benefit from including additional measurements in risk stratification models, questionnaire-only models predict prevalent and incident diabetes with high accuracy and clinical utility. By not being subject to the practical limitations associated with collecting physical measurements or biomarkers, a questionnaire-based tool comprises the first step towards identifying an initial high-risk population that could be referred for subsequent diagnostic or prognostic assessment in a primary care setting. At a sensitivity and specificity as high as 80%, we see that questionnaire-only models applied to the largest population we studied, with almost 180,000 White individuals in the UK Biobank training set for incidence prediction, would recommend follow-up for less than 40,000 individuals based on their eight-year T2D risk, and around 65,000 high-risk individuals with prevalent T2D (Supplementary Tables S5A and S5B). In the context of population health prevention programs, deploying more selective models brings about two advantages. On the one hand, it requires considerably fewer individuals to be screened to detect a substantial portion of high-risk individuals. On the other hand, in line with previous research, it has been shown that such programs are most effective when targeted at a specific outcome, such as T2D risk reduction, and when including high-risk individuals, as opposed to a non-stratified population.^32^ Based on our reclassification analyses, all models developed herein correctly reclassify predicted T2D cases and, in many instances, outperform the currently available models. Of note, our models have demonstrated significantly better net reclassification improvements and correctly reclassify more events when compared to available clinical tools. Specifically, when compared to FINDRISC, an additional 4,651 positive cases are correctly reclassified using our models per 40,000 events, reaching statistical significance. Likewise, for the comparisons with AUSDRISK, the respective number of positive cases that are correctly and significantly reclassified using our models is 3,155 per 20,000 events.

Eventually, translating the models presented in this proof of principle study into population health risk stratification tools for primary diabetes care is not without challenges. In fact, most digital health innovations fail to advance into clinical practice or fall short of their anticipated impact.^33^ This lack of adoption is often the result of a poor understanding of end-user needs and an inability to integrate the solution into current care frameworks.^33^ We built questionnaire-only models to predict and diagnose T2D with the intent that individuals could complete them, potentially digitally, without requiring invasive biomarker collection or a visit to primary care facilities. While not replacing a trained clinician's evaluation, a patient-centred tool would facilitate timely screening and reach a larger audience by eliminating the need for primary care visits in the first phase. Policymakers have been encouraged to focus on prevention and innovation to enable large-scale diabetes awareness programmes.^34^ For such initiatives, another possible challenge in applying questionnaire-based models at scale is to ensure that all questions are answered. Therefore, we limited the number of questions included to ten.

Overall, our study has several strengths and certain inherent limitations. First, this study represents the largest hitherto reporting on the performance and potential clinical utility of a questionnaire-based risk stratification model for prevalent and incident T2D in two biobanks and across multiple ethnicities. From a modelling perspective, this minimises the chances of overfitting and provides evidence of the model's validity. Second, we applied strict inclusion and exclusion criteria, thereby minimising the risk of including individuals with undiagnosed T2D. Third, we validated two widely non-laboratory clinical tools, FINDRISC and AUSDRISK, in all ethnic groups of the UK Biobank and externally in Lifelines, which provides a comprehensive benchmark for the performance of our models. On the other hand, as with all self-reported biobank data, ethnicity data may only be partially accurate. Specifically, the self-reported ethnic background can be influenced by individual perceptions, cultural and social factors, and may not always accurately reflect an individual's ancestry and levels of admixture. Additionally, the categories used to describe ethnicity can differ between countries, making it difficult to compare results across studies. Another potential limitation lies in the categorisation of “potentially undiagnosed” T2D. To try to minimise the risk of including individuals who may have clinically high, although not repeatedly, plasma glucose or HbA1c concentrations without confirmed T2D diagnosis, we set the plasma glucose exclusion threshold at above 7 mmol/L and the HbA1c exclusion threshold at above 48 mmol/mol. These thresholds may not be realistic or indicative for “potentially undiagnosed” T2D since plasma glucose values are sometimes obtained in a non-fasted state or may not be reproduced if repeatedly tested. Thus, excluding “potentially undiagnosed” cases of T2D might have impacted the performance of the models presented herein. Besides, for prevalence prediction, in our study, individuals are already aware of their diagnosis, and if the questionnaire models were to be prospectively applied, the answers of individuals knowing they have T2D might be different from those unaware of it (undiagnosed cases). Moreover, these questionnaires were administered as part of a volunteer-led biobank cohort whose participants tend to be relatively healthier or younger individuals, placing limitations around the age distribution to which they apply and potentially socioeconomic limitations. Lastly, due to the observational nature of this study, we cannot identify causal relationships between the variables included in the models and the predicted outcomes.

In conclusion, questionnaire-based ML models predict prevalent and incident T2D in multiple ethnicities with high accuracy and have the potential to enhance early diagnosis if deployed for population health screening in primary diabetes care. While biomarker-based models achieved enhanced performance, the questionnaire-only models produced significantly high and clinically useful predictions to be considered a valid alternative to these models and the challenges their large-scale deployment can pose. This is particularly important for populations of non-White ethnicity who are disproportionately impacted by T2D and regions with limited resources and access to primary diabetes care. While current prediction models show promise in diagnosing and predicting T2D, further research is needed to determine the effectiveness of these models in identifying undiagnosed type 2 diabetes. Specifically, a follow-up study is required using a cohort where undiagnosed cases can be correctly identified. This effectiveness should be validated in cohorts of different populations and ethnic makeups, as this may vary between these groups.

## Contributors

MK, SvD, JCF, DdV, and BHRW conceived and designed the study. MK interpreted the data and analyses, conducted the literature search, made the figures, and wrote the manuscript. PF accessed the data, conducted data cleaning, statistical analyses, made the figures, and wrote the manuscript. SvD contributed to data interpretation and wrote the manuscript. MK and SvD accessed and verified the underlying data and analyses. NS, ST, OT, YI, RHH, and BHRW contributed to writing and reviewing the manuscript. JCF and CSM contributed to advising, writing and reviewing the manuscript and interpreting the analyses. DdV contributed to writing, reviewing, and interpreting the analyses and creating the figures. DdV and BHRW worked in supervisory capacities and contributed equally to the work presented herein. MK has full access to all the data in the study and had final responsibility for the decision to submit for publication.

## Data sharing statement

Study data are available from UK Biobank and Lifelines but were used under license for the current study, which restricts their public availability. Data are, however, available from the authors upon reasonable request and when granted permission by the UK Biobank and Lifelines. All code is available and can be requested through the corresponding author.

## Declaration of interests

MK, NS, ST, OC, YI, and RHH have no conflict of interest to declare. PF, SvD, JCF, and DdV are employed by Ancora Health B.V. and own shares of Ancora Health B.V. BHRW sits on the medical advisory board of Ancora Health B.V. CSM has been a shareholder of and reports grants through his institution and personal consulting fees from Coherus Inc., AltrixBio, grants through his institution from Merck, and grants through his institution personal consulting fees from Novo Nordisk, reports personal consulting fees and support with research reagents from Ansh Inc., reports personal consulting fees from Genfit, Lumos, Amgen, Corcept, Intercept, 89Bio, AstraZeneca and Regeneron, reports support (educational activity meals at and through his institution) from Amarin, Novo Nordisk and travel support and fees from TMIOA, Elsevier, the California Walnut Commission, College Internationale Research Servier, and the Cardio Metabolic Health Conference; none of which is related to the work presented herein.
