# Supplementary material for: Effective questionnaire-based prediction models for type 2 diabetes across several ethnicities: a model development and validation study
Source: eClinicalMedicine. 2023 Sep 25;64:102235. doi: 10.1016/j.eclinm.2023.102235 (PMC10626169; doi:10.1016/j.eclinm.2023.102235)
Supplement: Supplementary Material [file mmc1.pdf]

Effective questionnaire-based prediction models for type 2 diabetes across several ethnicities: a model development and validation study

Supplementary

Table of Contents

Basic analysis *2*

Questionnaire models *5*

Diagnostic performance 5

Feature selection and correlation 6

Model performance 7

Questionnaire & Basic measurements models *8*

Diagnostic performance 8

Feature selection and correlation 9

Model performance 10

Risk stratification 11

Questionnaire, Basic measurements, and Biomarkers models *12*

Diagnostic performance 12

Feature importance 13

Model performance 14

Risk stratification 15

Overall model comparison *16*

Reclassification Analysis *20*

Basic analysis

Supplementary Table S1A. UK Biobank type 2 diabetes annotation.

**Diagnosis**		**Age of diagnosis**	
**Data-Field**	**Name**	**Data-Field**	**Name**
20002	Non-cancer illness code, self-reported	20009	Interpolated Age of participant when non-cancer illness first diagnosed
2443	Diabetes diagnosed by doctor	2976	Age diabetes diagnosed
41271	Diagnoses – ICD9	41281	Date of first in-patient diagnosis - ICD9
41270	Diagnoses - ICD10	41280	Date of first in-patient diagnosis - ICD10

Supplementary Table S1B. Lifelines type 2 diabetes annotation.

**Prevalent diagnosis**		**Incident diagnosis**	
**Variable**	**Age of diagnosis**	**Variable**	**Age of diagnosis**
diabetes_type_adu_q_1 == 2	diabetes_startage_adu_q_1	t2d_followup_adu_q_1	Manually calculated by MEAN (age at assessment when diagnosis reported, age previous assessment). If diabetes_followup_adu_q_1 was answered before, the age at this assessment was used instead.
diabetes_type_adu_q_2 == 2	diabetes_presence_adu_q_2_a		

Supplementary Table S2. Mapped features between the UK Biobank and Lifelines. For ordinal UK Biobank features, numeric data values were used· Subsequently, each category was one hot encoded; these feature names are depicted by "*category* -- *UK Biobank feature*_0.0".

**UK Biobank feature**	**Lifelines feature**	**Notes**
**Questionnaire features**		
Age of attending assessment centre_0.0	median(AGE 1a)	In the UK Biobank, more specific ages of attending the assessment centre were calculated based on the date of attending the assessment centre minus the birth year/month of the participant.
Alcohol intake frequency_0.0	ffqh_alcohol_adu_q_27	Alcohol intake categories did not match exactly between UK Biobank and Lifelines. The Lifelines categories were therefore mapped as closely as possible to the UK Biobank categories.
Aspirin -- Medication for pain relief, constipation, heartburn_0.0	otc_painfever_adu_q_1	
Body mass index (BMI)_0.0	bodyweight_kg_all_m_1	

Bread intake_0.0	ffqh_breadproducts_adu_q_39	
CAD before first assessment. Annotated based on heart attack, PTCA, CABG, triple heart bypass, based on self report, OPCS4, ICD9 and ICD10	heartattack_presence_adu_q_2, heartattack_presence_adu_q_1, angioplasty_bypass_adu_q_1	
Blood pressure medication -- MERGED 6153 6177_0.0	hypertension_treatment_adu_q_1 == 3 or 4	In the UK Biobank, blood pressure medication was annotated by merging data fields 6153 and 6177.
Cholesterol lowering medication -- MERGED 6153 6177_0.0	highcholesterol_treatment_adu_q_1 == 3 or 4	In the UK Biobank, cholesterol lowering medication was annotated by merging data fields 6153 and 6177.
Pack years of smoking_0.0	packyears_cumulative_adu_c_2	In the UK Biobank, NA's were replaced with 0.
Unable to work because of sickness or disability -- Current employment status_0.0	employment_stopped_adu_q_1_d == 2	
Yes - lost weight -- Weight change compared with 1 year ago_0.0	bodyweight_highest_adu_q_1_a - bodyweight_kg_all_m_1 > 5	
Yes - gained weight -- Weight change compared with 1 year ago_0.0	bodyweight_lowest_adu_q_1_a - bodyweight_kg_all_m_1 > 5	
Number of treatments/medications taken MAX 12 excl insulin_0.0	N atc_code_adu_c_01-32 - (diabetes_treatment_adu_q_1 == 3 or 4)	In the UK Biobank, values above 12 for "Number of treatments/medications taken" were put on 12 to remove outliers. The number 12 was manually chosen as the cut-off based on a histogram of the values. Subsequently, for all insulin users, 1 point was subtracted.
Diabetes illness of father and/or mother_0.0	t2d_father_fam_q_1 AND/OR t2d_mother_fam_q_1	In the UK Biobank, consisting of Diabetes – Illness of father_0.0 AND/OR Diabetes – Illness of mother_0.0
Diabetes -- Illnesses of siblings_0.0	t2d_sibling_fam_q_1	
**Basic measurements features**		
Waist circumference_0.0	circumference_waist_all_m_1	
Pulse rate, automated reading MEAN_0.0	bpavg_pulse_all_m_1	In the UK Biobank, automated and manual reading were combined, and the mean of the two measured pulse rates was taken.
Diastolic blood pressure MEAN_0.0	bpavg_diastolic_all_m_1	In the UK Biobank, automated and manual readings were combined, and the mean of all blood pressure measurements was taken.
**Biomarker features**		
Glucose_0.0	glucose_result_all_m_1	

Glycated haemoglobin (HbA1c)_0.0	hba1cconc_result_all_m_1	
Cholesterol_0.0	cholesterol_result_all_m_1	

Supplementary Table S3. Ethnic group formations in the UK Biobank.

**Ethnic group**	**UK Biobank entries (Data-Field 21000: Ethnic background)**
White	White, British, Irish, Any other white background
South Asian	Indian, Pakistani, Bangladeshi
Caribbean	Caribbean, White and Black Caribbean
East Asian	Asian or Asian British, Chinese, White and Asian, Any other Asian background
Black	Black or Black British, African, White and Black African, Any other Black background
Other	Other ethnic group, Do not know, Prefer not to answer, Mixed, Any other mixed background

Supplementary Table S4. Ethnic makeup of the Lifelines cohort as established at baseline.

**Ethnicity**	**Frequency**	**Percentage**
White/East and West European	121,199	98%
White/Mediterranean or Arabic	432	0·3%
Black	201	0·2%
Asian	613	0·5%
Other, namely	1,248	1%
Total reported ethnicities	123,733	100%
No ethnicity reported	44,512	NA

Questionnaire models

Diagnostic performance

Supplementary Table S5A. Prevalent type 2 diabetes prediction optimised with Youden's method. High- and low-risk groups were created based on the same probability thresholds as presented in the last column; Positive Predictive Value (PPV), Negative Predictive Value (NPV).

Population	N	Low-risk	High-risk	Sensitivity (95% CI)	Specificity (95% CI)	PPV (95% CI)	NPV (95% CI)	Threshold
**White**	317779	251595	66184	83 (83-84)	83 (83-83)	24 (24-24)	99 (99-99)	0·053
**South Asian**	5605	3688	1917	80 (78-82)	80 (79-81)	55 (53-57)	93 (92-94)	0·065
**Caribbean**	2960	2216	744	80 (76-84)	85 (84-86)	50 (46-54)	96 (95-97)	0·068
**East Asian**	2907	2270	637	75 (70-79)	86 (85-87)	44 (41-48)	96 (95-97)	0·065
**Black**	2151	1573	578	75 (71-80)	82 (80-84)	45 (41-49)	95 (93-96)	0·061
**Other**	4621	3540	1081	80 (77-83)	84 (83-86)	42 (39-45)	97 (96-97)	0·065
**Lifelines**	91736	78380	13356	83 (81-84)	87 (87-87)	11 (10-11)	100 (100-100)	0·048

Supplementary Table S5B. Incident type 2 diabetes prediction optimised with Youden's method. High- and low-risk groups were created based on the same probability thresholds as presented in the last column; Positive Predictive Value (PPV), Negative Predictive Value (NPV).

Population	N	Low-risk	High-risk	Sensitivity (95% CI)	Specificity (95% CI)	PPV (95% CI)	NPV (95% CI)	Threshold
**White**	179740	140404	39336	79 (78-80)	80 (80-80)	10 (10-10)	99 (99-99)	0·027
**South Asian**	2408	1568	840	81 (75-86)	69 (67-71)	19 (16-22)	98 (97-98)	0·014
**Caribbean**	1559	1259	300	76 (65-84)	84 (82-86)	22 (17-27)	98 (97-99)	0·038
**East Asian**	1019	756	263	80 (68-89)	78 (75-80)	19 (14-24)	98 (97-99)	0·013
**Black**	3435	2288	1147	98 (89-100)	68 (66-69)	4 (3-5)	100 (100-100)	0·012
**Other**	3363	2635	728	79 (70-86)	80 (79-82)	12 (9-14)	99 (99-99)	0·027
**Lifelines**	32686	23739	8947	75 (71-78)	73 (73-74)	5 (4-5)	99 (99-99)	0·011

Feature selection and correlation

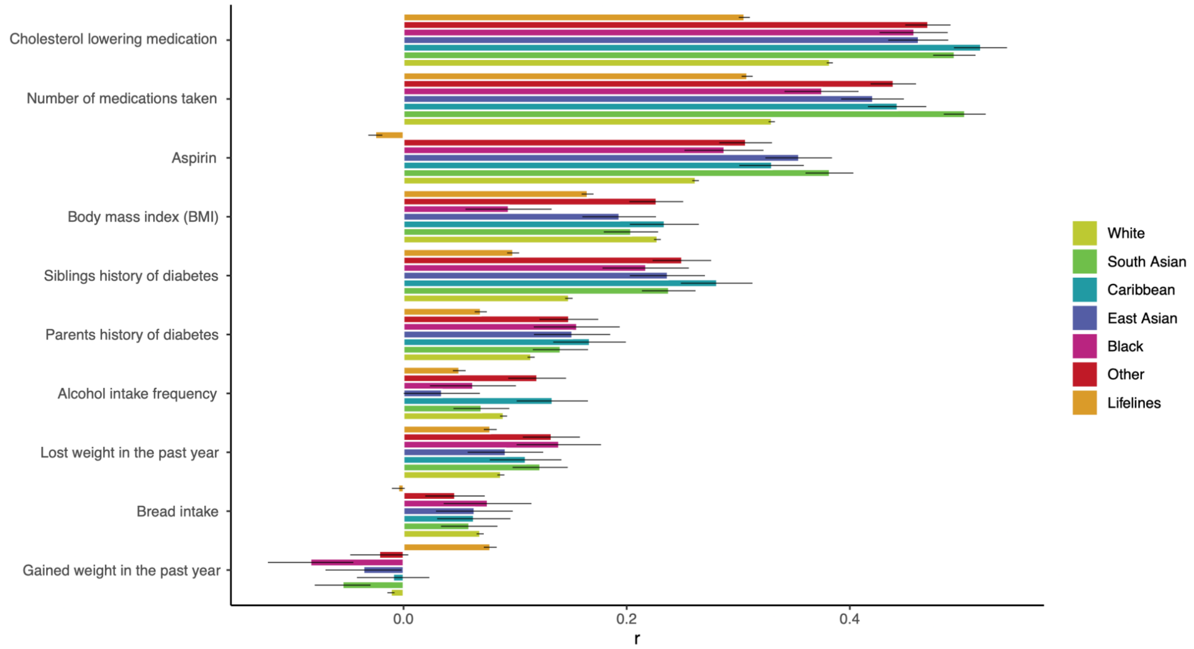

Supplementary Figure S2A. The correlation between each feature and prevalent type 2 diabetes for each population. Higher r values indicate positive correlations, while negative r values show negative correlations between both variables.

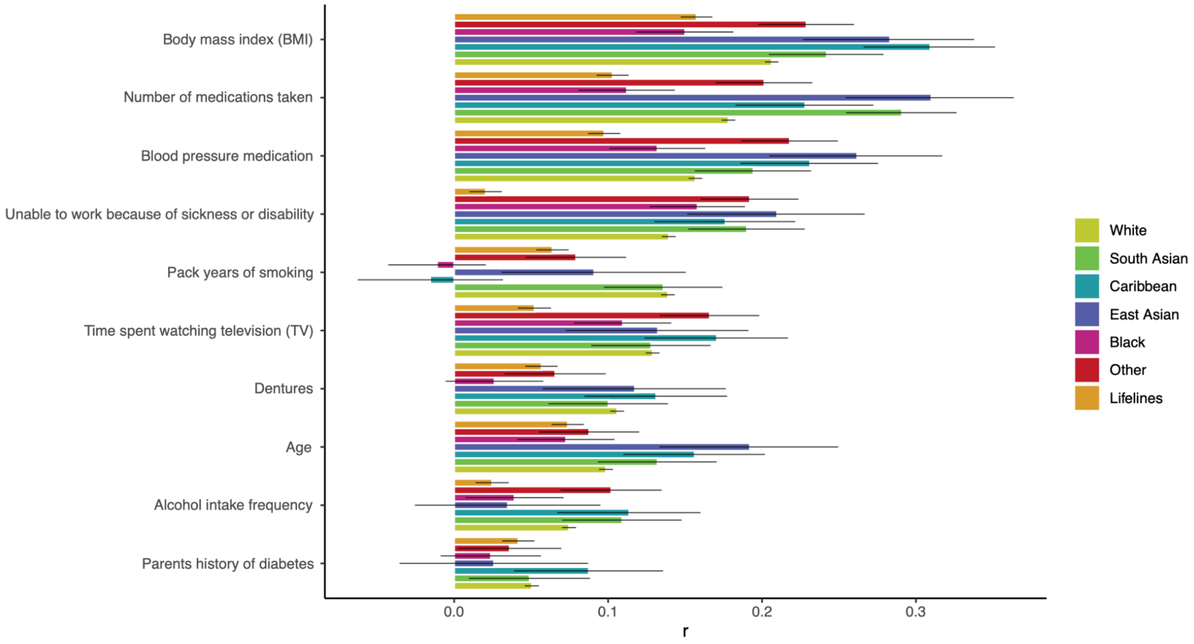

Supplementary Figure S2B. The correlation between each feature and incident type 2 diabetes for each population. Higher r values indicate positive correlations, while negative r values show negative correlations between both variables.

Model performance

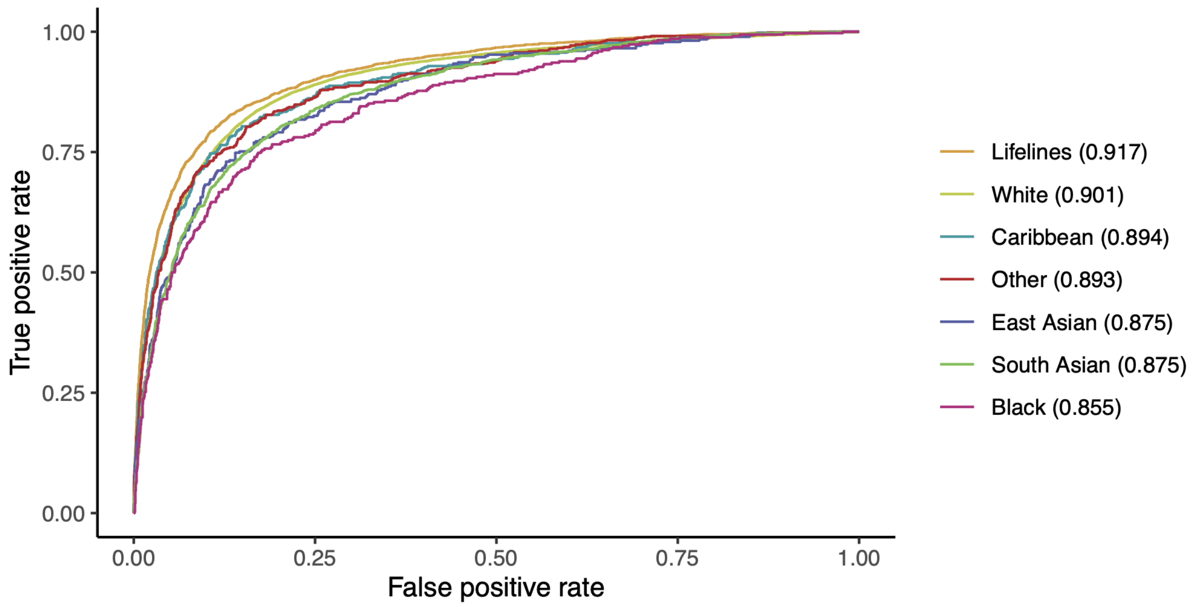

Supplementary Figure S3A. AUC scores for the questionnaire-based prevalence prediction models.

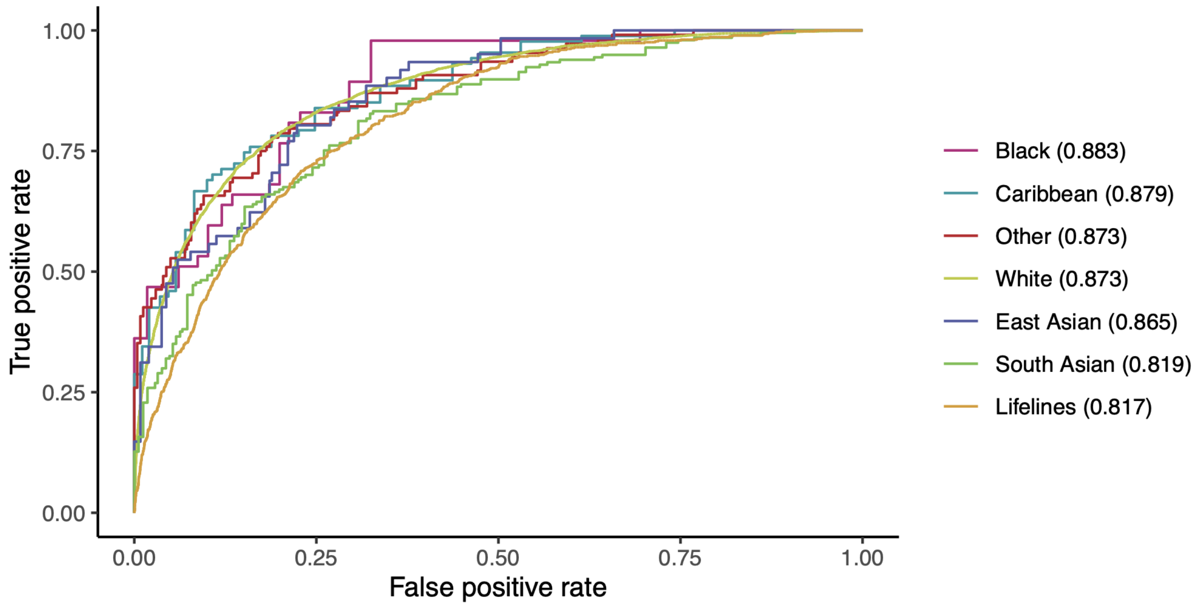

Supplementary Figure S3B. AUC scores for the questionnaire-based incidence prediction models.

Questionnaire & Basic measurements models

Diagnostic performance

Supplementary Table S6A. Prevalent type 2 diabetes prediction optimised with Youden's method. High- and low-risk groups were created based on the same probability thresholds as presented in the last column; Positive Predictive Value (PPV), Negative Predictive Value (NPV).

Population	N	Low-risk	High-risk	Sensitivity (95% CI)	Specificity (95% CI)	PPV (95% CI)	NPV (95% CI)	Threshold
**White**	321178	258449	62729	83 (82-83)	85 (84-85)	26 (25-26)	99 (99-99)	0·059
**South Asian**	5792	3864	1928	81 (79-83)	81 (80-82)	57 (55-59)	93 (93-94)	0·082
**Caribbean**	3061	2403	658	80 (76-83)	90 (88-91)	59 (56-63)	96 (95-97)	0·104
**East Asian**	2971	2255	716	79 (74-82)	84 (83-86)	43 (39-47)	96 (95-97)	0·061
**Black**	2252	1529	723	82 (78-86)	77 (75-79)	41 (37-44)	96 (95-97)	0·045
**Other**	4777	3862	915	77 (73-80)	89 (88-90)	49 (46-52)	97 (96-97)	0·096
**Lifelines**	93263	79599	13664	81 (80-83)	87 (86-87)	11 (11-12)	100 (100-100)	0·103

Supplementary Table S6B. Incident type 2 diabetes prediction optimised with Youden's method. High- and low-risk groups were created based on the same probability thresholds as presented in the last column; Positive Predictive Value (PPV), Negative Predictive Value (NPV).

Population	N	Low-risk	High-risk	Sensitivity (95% CI)	Specificity (95% CI)	PPV (95% CI)	NPV (95% CI)	Threshold
**White**	183185	145425	37760	78 (77-79)	81 (81-81)	11 (11-11)	99 (99-99)	0·029
**South Asian**	2444	1573	871	80 (74-85)	68 (66-70)	19 (16-22)	97 (96-98)	0·015
**Caribbean**	1670	1182	488	88 (79-94)	74 (72-76)	16 (13-20)	99 (98-100)	0·017
**East Asian**	1034	752	282	85 (74-92)	77 (74-79)	20 (15-25)	99 (98-99)	0·010
**Black**	3644	2586	1058	89 (77-96)	72 (70-73)	4 (3-5)	100 (100-100)	0·019
**Other**	3438	2513	925	82 (73-88)	75 (73-76)	10 (8-12)	99 (99-99)	0·022
**Lifelines**	32686	20914	11772	86 (83-88)	65 (64-65)	4 (4-5)	100 (100-100)	0·012

Feature selection and correlation

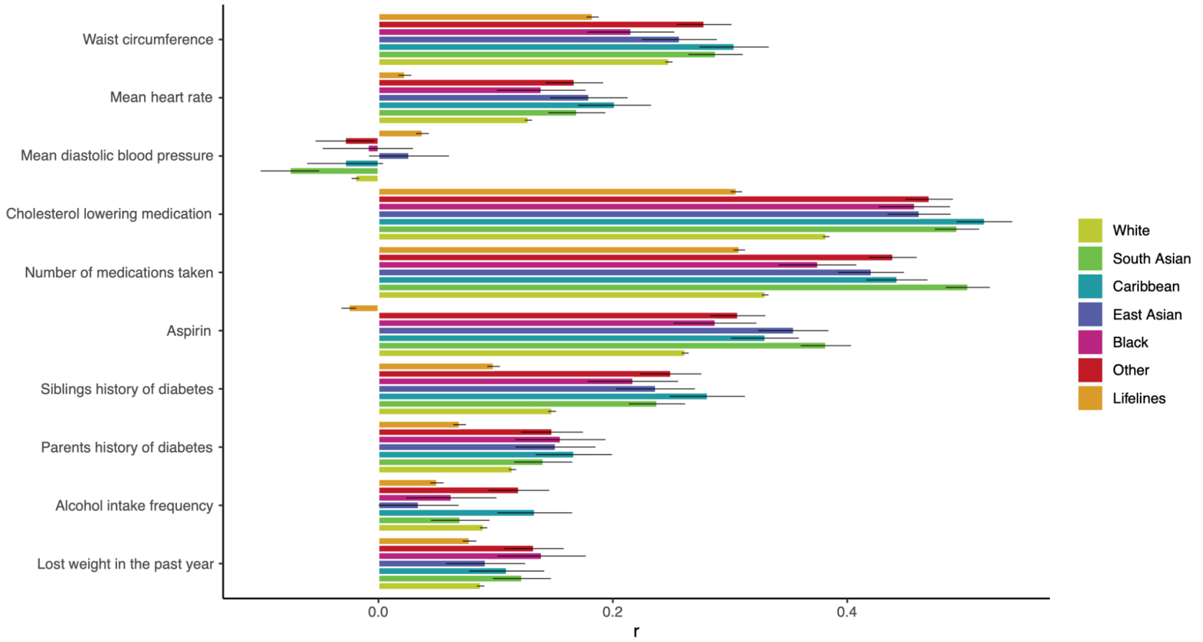

Supplementary Figure S4A. The correlation between each feature and prevalent type 2 diabetes for each population. Higher r values indicate positive correlations, while negative r values show negative correlations between both variables.

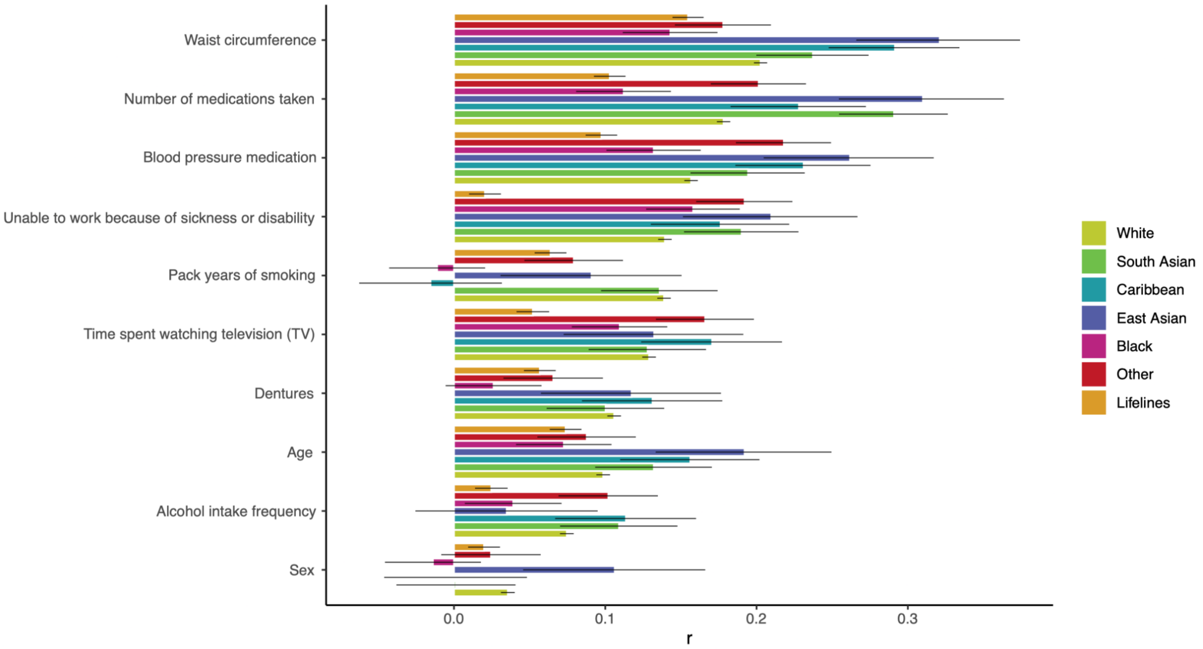

Supplementary Figure S4B. The correlation between each feature and incident type 2 diabetes for each population. Higher r values indicate positive correlations, while negative r values show negative correlations between both variables.

Model performance

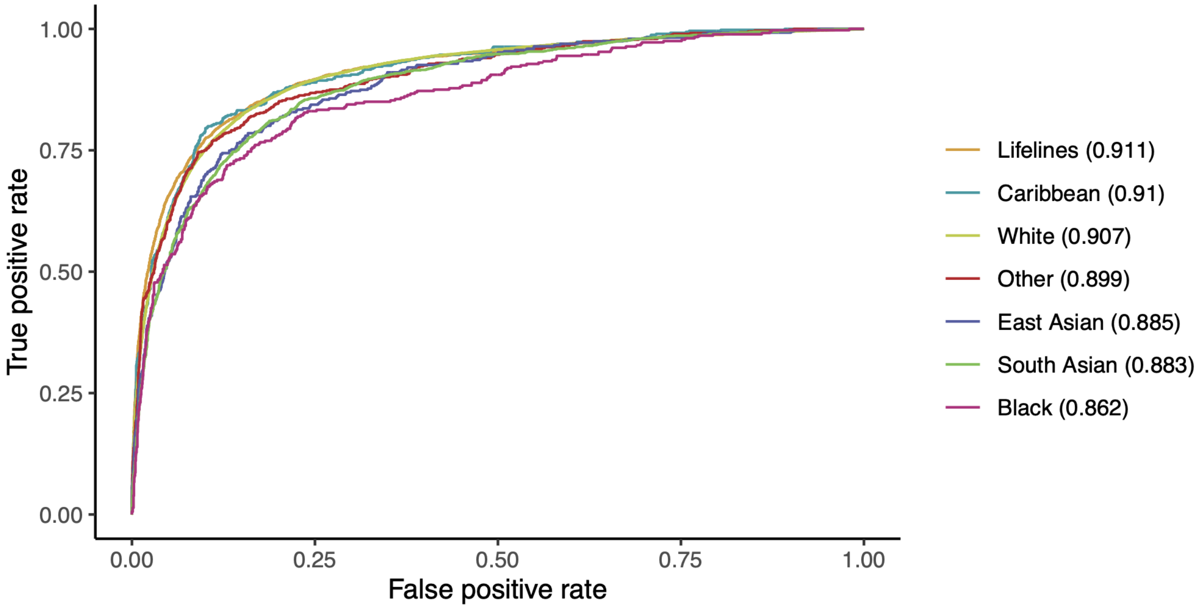

Supplementary Figure S5A. AUC scores for the questionnaire & basic measurements-based prevalence prediction models.

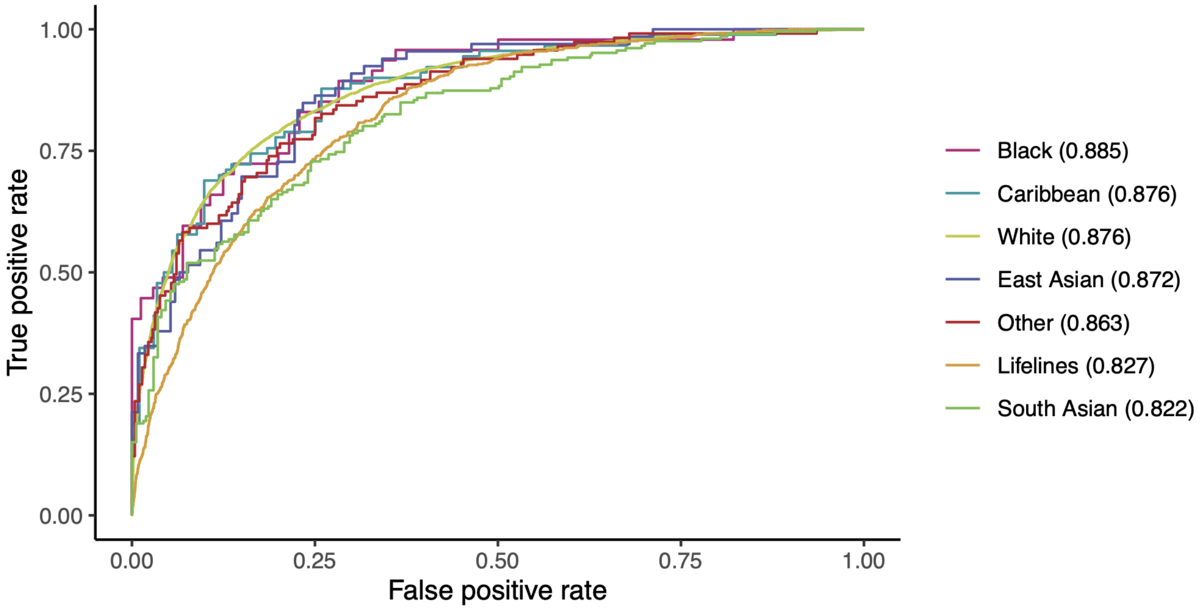

Supplementary Figure S5B. AUC scores for the questionnaire & basic measurements-based incidence prediction models.

Risk stratification

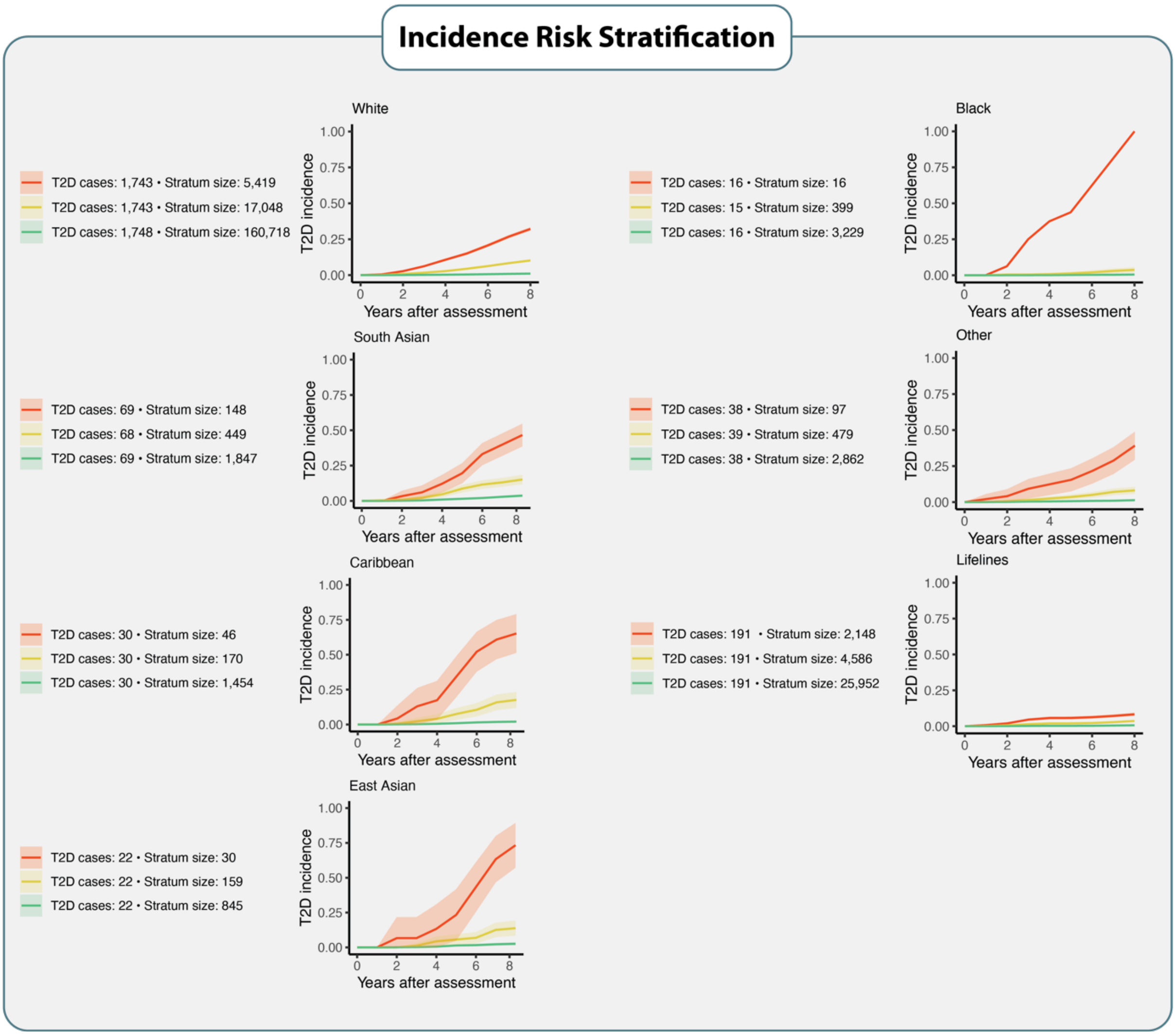

Supplementary Figures S6. Risk identification for developing T2D per ethnic group. Every ethnic group is separated into three risk strata, according to the individuals' risk of incident T2D (high risk = red, medium risk = yellow, low risk = green), with each risk stratum containing 33% of all T2D cases. The x-axis represents the interval of years between the biobank entry and the moment of receiving a diagnosis of T2D. The y-axis represents the incidence of T2D. The stronger-coloured lines represent the average T2D incidence within each risk stratum, and the lighter-coloured bands around the lines show the 95% CI. T2D cases correspond to the total number of T2D incident cases within each risk stratum. Stratum size corresponds to the number of individuals within each risk stratum. Stratum sizes show how many individuals must be screened to identify 33% of all T2D cases within each risk-stratum.

Questionnaire, Basic measurements, and Biomarkers models

Diagnostic performance

Supplementary Table S7A. Prevalent type 2 diabetes prediction optimised with Youden's method. High- and low-risk groups were created based on the same probability thresholds as presented in the last column; Positive Predictive Value (PPV), Negative Predictive Value (NPV).

Population	N	Low-risk	High-risk	Sensitivity (95% CI)	Specificity (95% CI)	PPV (95% CI)	NPV (95% CI)	Threshold
**White**	318198	288426	29772	88 (88-89)	95 (95-95)	48 (47-48)	99 (99-99)	0·063
**South Asian**	5532	4310	1222	89 (87-91)	94 (93-95)	78 (75-80)	97 (97-98)	0·153
**Caribbean**	2906	2521	385	84 (80-88)	96 (95-97)	72 (67-77)	98 (97-98)	0·252
**East Asian**	2884	2458	426	88 (84-91)	94 (93-95)	62 (57-66)	99 (98-99)	0·103
**Black**	2128	1758	370	85 (80-90)	91 (89-92)	52 (47-57)	98 (97-99)	0·111
**Other**	4674	4068	606	88 (84-91)	95 (94-96)	66 (62-69)	99 (98-99)	0·129
**Lifelines**	112745	105903	6842	93 (92-94)	96 (96-96)	30 (29-31)	100 (100-100)	0·114

Supplementary Table S7B. Incident type 2 diabetes prediction optimised with Youden's method. High- and low-risk groups were created based on the same probability thresholds as presented in the last column; Positive Predictive Value (PPV), Negative Predictive Value (NPV).

Population	N	Low-risk	High-risk	Sensitivity (95% CI)	Specificity (95% CI)	PPV (95% CI)	NPV (95% CI)	Threshold
**White**	179538	149879	29659	84 (82-85)	85 (85-86)	14 (14-15)	99 (99-99)	0·028
**South Asian**	2353	1772	581	88 (83-93)	81 (79-83)	30 (27-34)	99 (98-99)	0·034
**Caribbean**	1630	1341	289	91 (83-96)	86 (85-88)	27 (22-33)	99 (99-100)	0·057
**East Asian**	991	836	155	86 (75-93)	89 (87-91)	36 (29-44)	99 (98-100)	0·028
**Black**	3577	3050	527	76 (61-87)	86 (85-87)	7 (5-9)	100 (99-100)	0·086
**Other**	3394	2912	482	89 (81-94)	88 (87-89)	21 (17-25)	100 (99-100)	0·034
**Lifelines**	32218	26388	5830	82 (78-85)	83 (83-83)	8 (7-9)	100 (100-100)	0·053

Feature importance

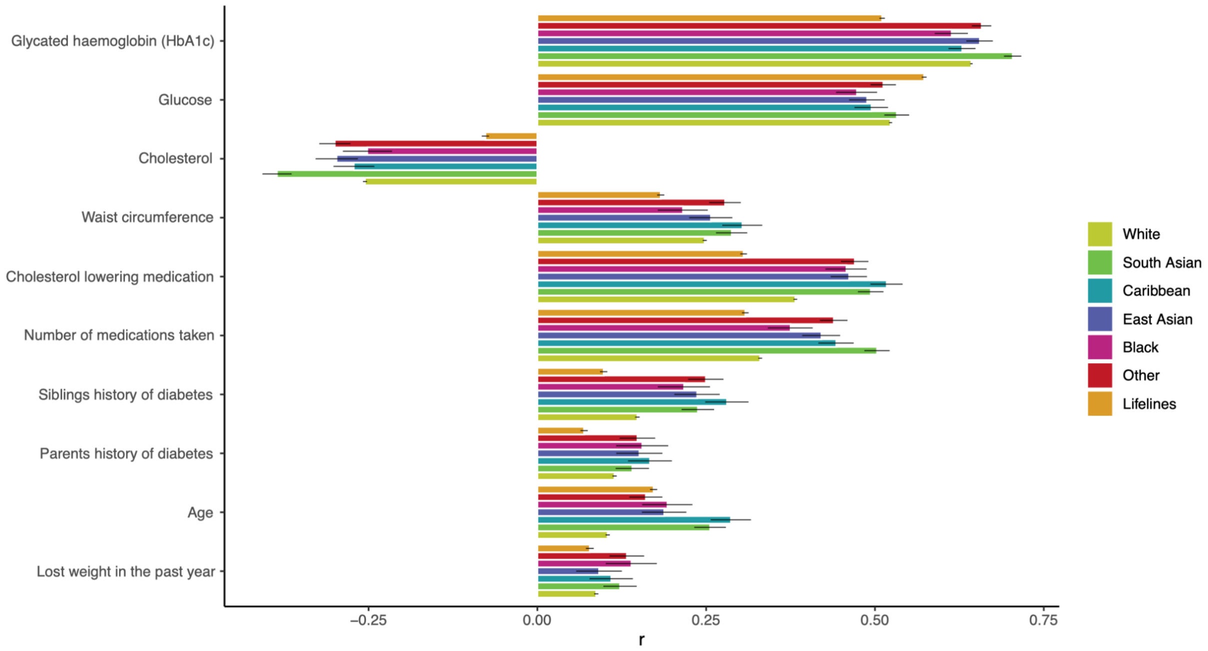

Supplementary Figure S7A. AUC scores for the questionnaire, basic measurements, and biomarkers-based prevalence prediction models.

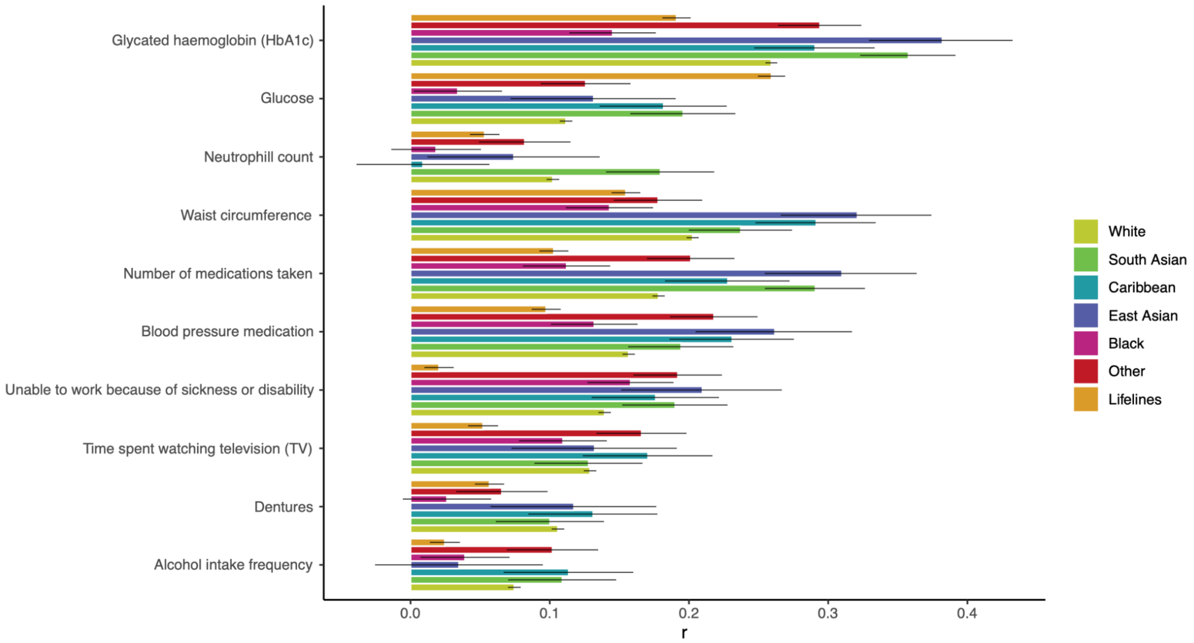

Supplementary Figure S7B. AUC scores for the questionnaire, basic measurements, and biomarkers-based incidence prediction models.

Model performance

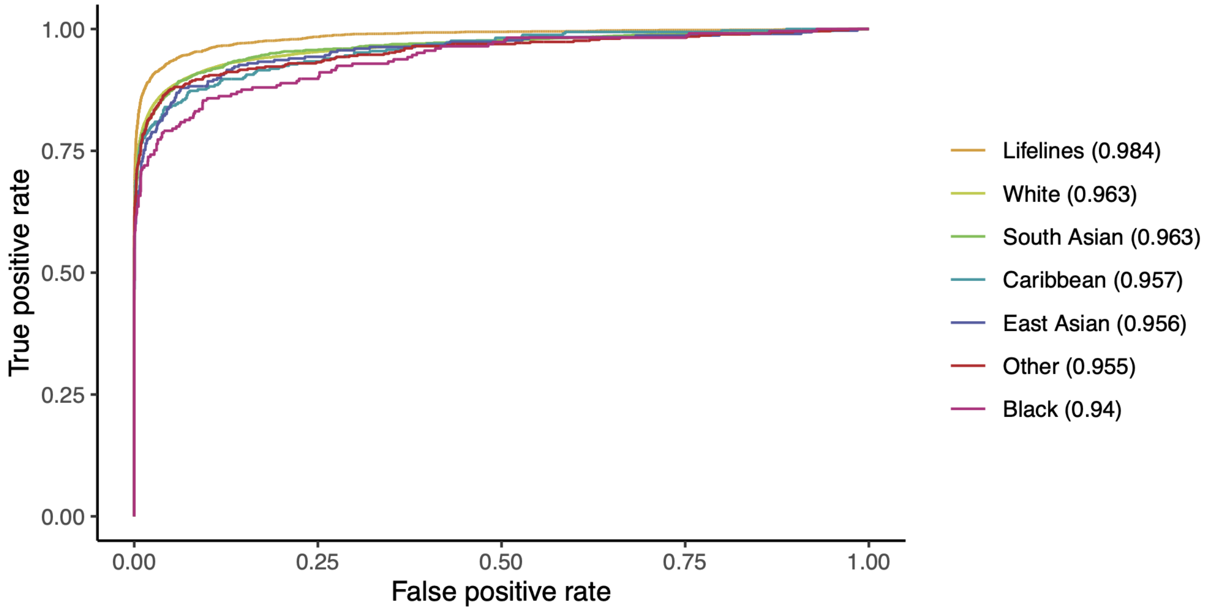

Supplementary Figure S8A. AUC scores for the questionnaire, basic measurements & biomarkers-based prevalence prediction models.

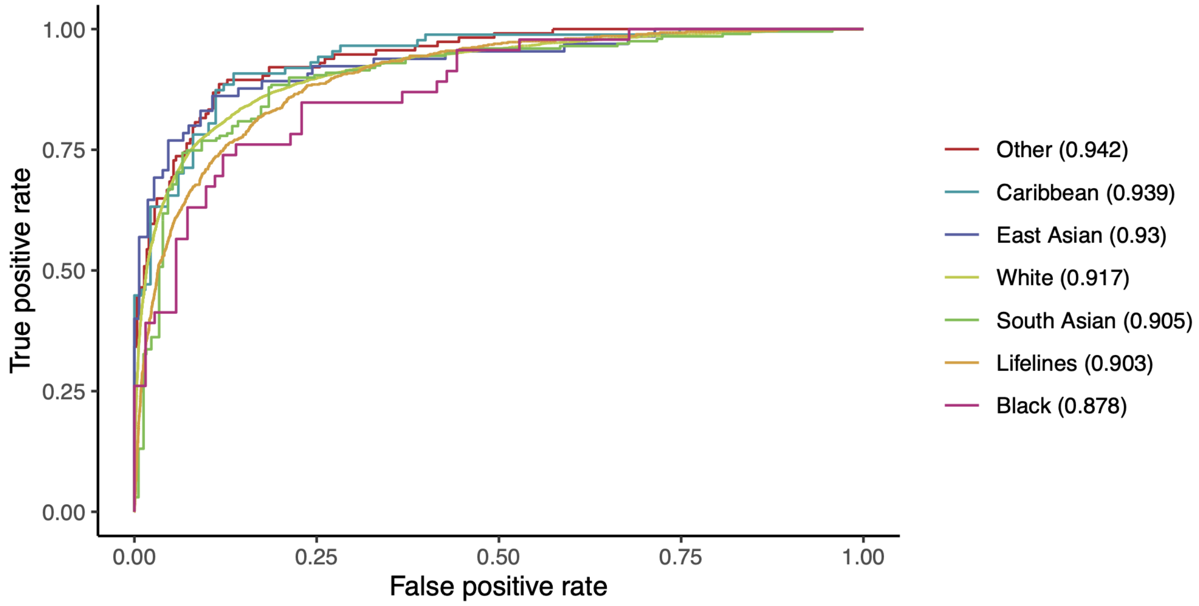

Supplementary Figure S8B. AUC scores for the questionnaire, basic measurements & biomarkers-based incidence prediction models.

Risk stratification

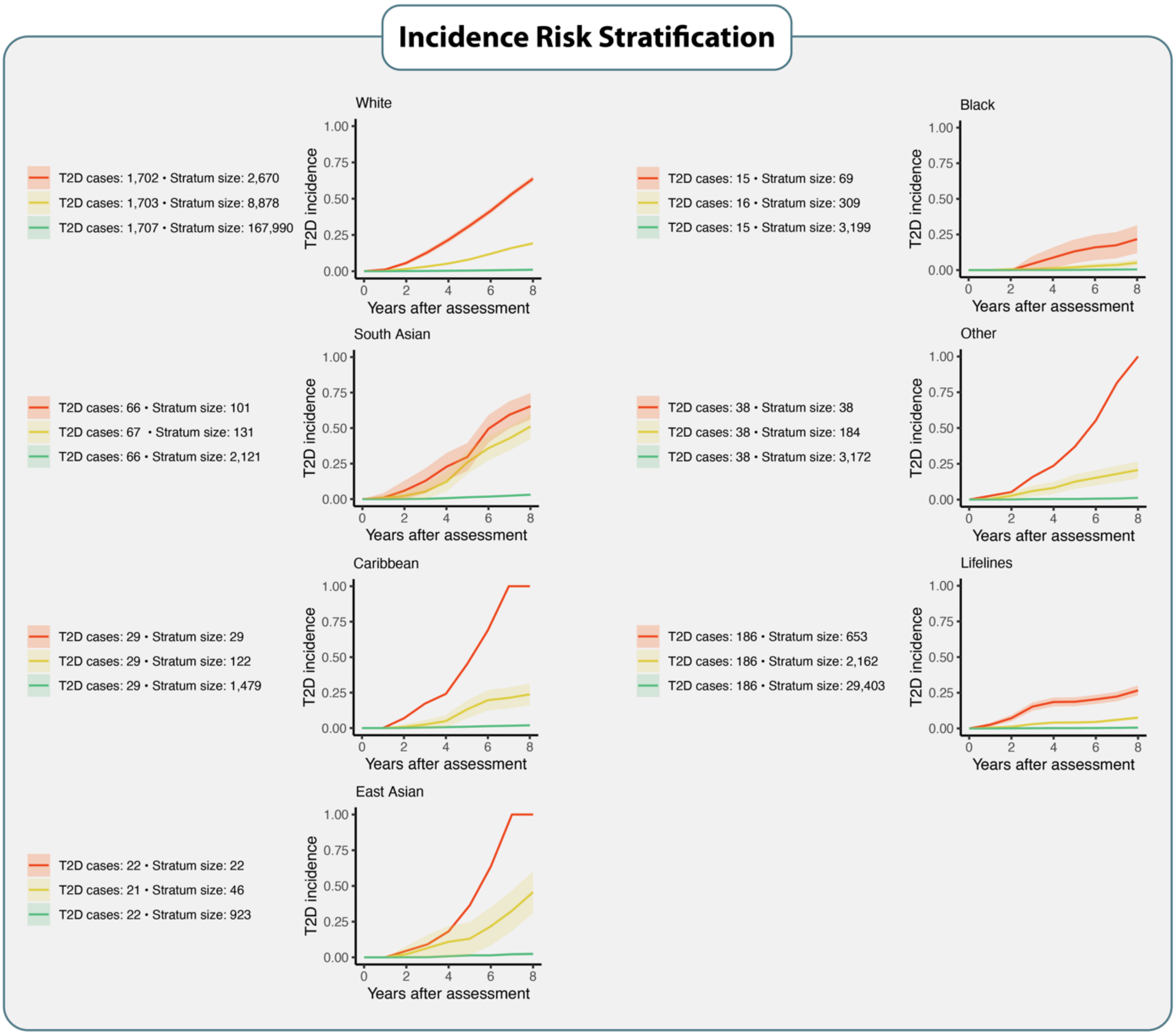

Supplementary Figures S9. Risk identification for developing T2D per ethnic group. Every ethnic group is separated into three risk strata, according to the individuals' risk of incident T2D (high risk = red, medium risk = yellow, low risk = green), with each risk stratum containing 33% of all T2D cases. The x-axis represents the interval of years between the biobank entry and the moment of receiving a diagnosis of T2D. The y-axis represents the incidence of T2D. The stronger-coloured lines represent the average T2D incidence within each risk stratum, and the lighter-coloured bands around the lines show the 95% CI. T2D cases correspond to the total number of T2D incident cases within each risk stratum. Stratum size corresponds to the number of individuals within each risk stratum. Stratum sizes show how many individuals must be screened to identify 33% of all T2D cases within each risk-stratum.

Overall model comparison

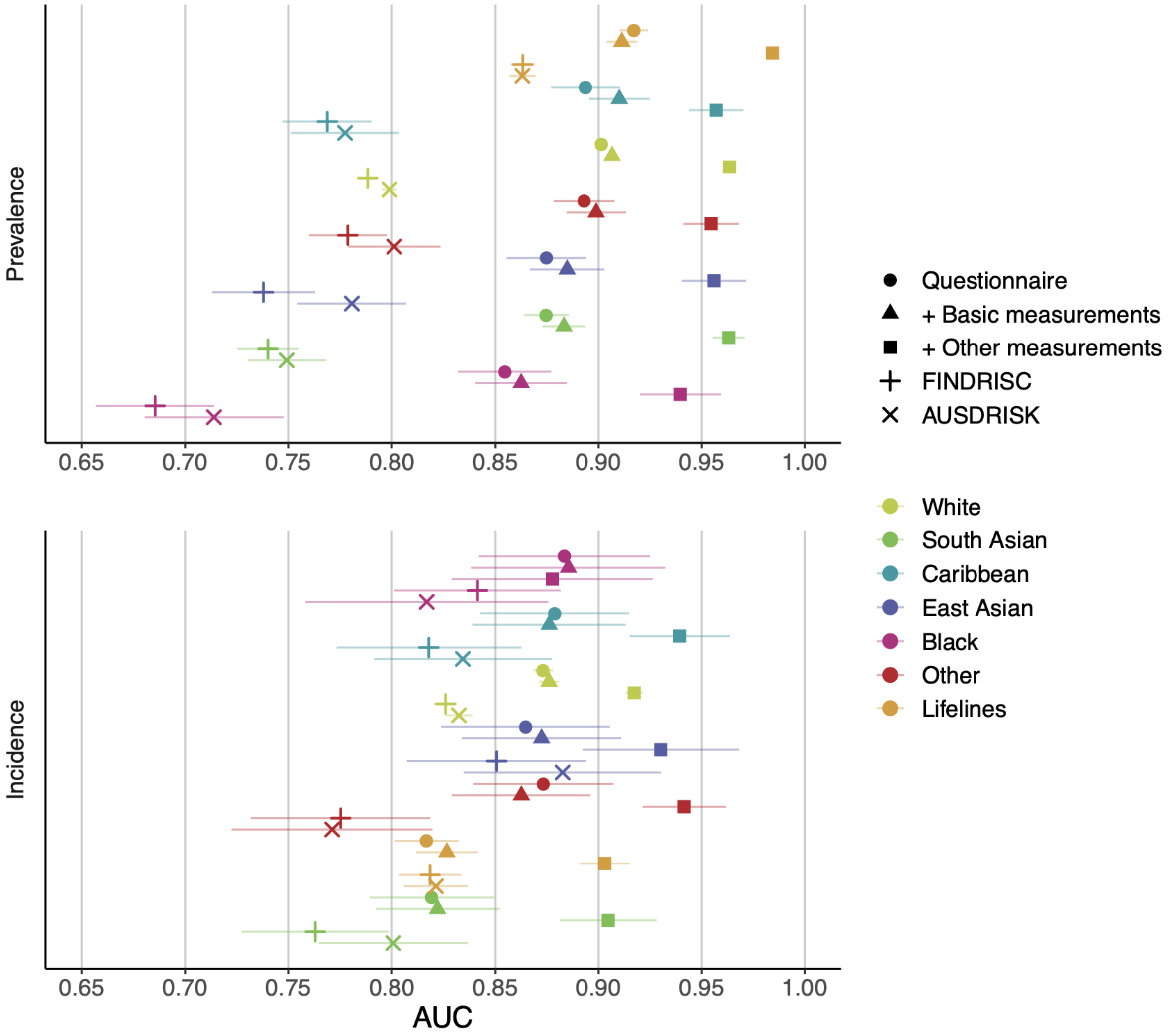

Supplementary Figure S10. All models' performances. Each colour-symbol combination refers to a specific model and population. The AUC and 95% CI are shown for all models.

Supplementary Table S8A. Prevalence prediction comparison between our models.

Population	Questionnaire-only VS Questionnaire & Basic measurements (P-value)	Questionnaire, Basic measurements & Biomarkers VS Questionnaire-only (P-value)	Questionnaire, Basic measurements & Biomarkers VS Questionnaire & Basic measurements (P-value)
**White**	<0·001	<0·001	<0·001
**South Asian**	<0·001	<0·001	<0·001
**Caribbean**	<0·001	<0·001	<0·001
**East Asian**	0·04	<0·001	<0·001
**Black**	0·04	<0·001	<0·001
**Other**	0·06	<0·001	<0·001
**Lifelines**	0·03	<0·001	<0·001

Supplementary Table S8B. Incidence prediction comparison between our models.

Population	Questionnaire-only VS Questionnaire & Basic measurements (P-value)	Questionnaire, Basic measurements & Biomarkers VS Questionnaire-only (P-value)	Questionnaire, Basic measurements & Biomarkers VS Questionnaire & Basic measurements (P-value)
**White**	0·01	<0·001	<0·001
**South Asian**	0·8	<0·001	<0·001
**Caribbean**	0·4	<0·001	<0·001
**East Asian**	0·1	0·002	0·002
**Black**	0·6	0·8	0·8
**Other**	0·2	<0·001	<0·001
**Lifelines**	<0·001	<0·001	<0·001

Supplementary Table S9A. Prevalence prediction comparison between our models and the clinical risk prediction tools AUSDRISK, FINDRISC.

Population	Model type	Models VS AUSDRISK (P-value)	Models VS FINDRISC (P-value)
**Black**	Questionnaire-only	<0·001	<0·001
**Other**	Questionnaire-only	<0·001	<0·001
**East Asian**	Questionnaire-only	<0·001	<0·001
**Caribbean**	Questionnaire-only	<0·001	<0·001
**South Asian**	Questionnaire-only	<0·001	<0·001
**White**	Questionnaire-only	<0·001	<0·001
**Lifelines**	Questionnaire-only	<0·001	<0·001
**Black**	Questionnaire & basic measurements	<0·001	<0·001
**Other**	Questionnaire & basic measurements	<0·001	<0·001
**East Asian**	Questionnaire & basic measurements	<0·001	<0·001
**Caribbean**	Questionnaire & basic measurements	<0·001	<0·001
**South Asian**	Questionnaire & basic measurements	<0·001	<0·001
**White**	Questionnaire & basic measurements	<0·001	<0·001
**Lifelines**	Questionnaire & basic measurements	<0·001	<0·001
**Black**	Questionnaire, basic measurements & biomarkers	<0·001	<0·001
**Other**	Questionnaire, basic measurements & biomarkers	<0·001	<0·001
**East Asian**	Questionnaire, basic measurements & biomarkers	<0·001	<0·001
**Caribbean**	Questionnaire, basic measurements & biomarkers	<0·001	<0·001
**South Asian**	Questionnaire, basic measurements & biomarkers	<0·001	<0·001
**White**	Questionnaire, basic measurements & biomarkers	<0·001	<0·001
**Lifelines**	Questionnaire, basic measurements & biomarkers	<0·001	<0·001

Supplementary Table S9B. Incidence prediction comparison between our models and the clinical risk prediction tools AUSDRISK, FINDRISC.

Population	Model type	Models VS AUSDRISK (P-value)	Models VS FINDRISC (P-value)
**Lifelines**	Questionnaire-only	0·4	0·5
**South Asian**	Questionnaire-only	0·04	<0·001
**East Asian**	Questionnaire-only	0·4	0·4
**White**	Questionnaire-only	<0·001	<0·001
**Other**	Questionnaire-only	<0·001	<0·001
**Caribbean**	Questionnaire-only	0·1	<0·001
**Black**	Questionnaire-only	0·2	0·2
**Lifelines**	Questionnaire & basic measurements	0·7	0·01
**South Asian**	Questionnaire & basic measurements	0·06	<0·001
**East Asian**	Questionnaire & basic measurements	0·6	0·08
**White**	Questionnaire & basic measurements	<0·001	<0·001
**Other**	Questionnaire & basic measurements	<0·001	<0·001
**Caribbean**	Questionnaire & basic measurements	0·5	0·006
**Black**	Questionnaire & basic measurements	0·05	0·1
**Lifelines**	Questionnaire, basic measurements & biomarkers	<0·001	<0·001
**South Asian**	Questionnaire, basic measurements & biomarkers	<0·001	<0·001
**East Asian**	Questionnaire, basic measurements & biomarkers	0·1	<0·001
**White**	Questionnaire, basic measurements & biomarkers	<0·001	<0·001
**Other**	Questionnaire, basic measurements & biomarkers	<0·001	<0·001
**Caribbean**	Questionnaire, basic measurements & biomarkers	<0·001	<0·001
**Black**	Questionnaire, basic measurements & biomarkers	0·1	0·2

Reclassification Analysis

Supplementary Table S10A. Reclassification analysis comparing our questionnaire-based model to FINDRISC and AUSDRISK. Reclassification events % correspond to our models' net percentage of reclassified individuals with T2D compared to the clinically established tools. Reclassification of events per 10,000 events corresponds to the net number of T2D cases reclassified when screening 10,000 cases. Positive reclassification events indicate that our models correctly reclassify more cases than the other two clinical tools, whereas negative events indicate the opposite. Reclassification percentages (%) are represented along with the 95% CI.

Risk model	Ethnicity	N high/low risk	Net Reclassification Improvement (NRI)	NRI p-value	Reclassification events %	Reclassification events p-value	Reclassification non-events %	Reclassification non-events p-value
**FINDRISC**	White	39,183/139,959	0·066 (0·054 - 0·078)	<0·001	6·4 (5·2 - 7·6)	<0·001	0·2 (0 - 0·4)	0·04
**FINDRISC**	Black	1,146/2,288	0·022 (-0·052 - 0·097)	0·6	2·2 (-5·2 - 9·5)	0·6	0 (-1·2 - 1·2)	1
**FINDRISC**	Caribbean	300/1,259	0·134 (0·043 - 0·225)	0·004	12·6 (3·7 - 21·5)	0·005	0·7 (-1·1 - 2·6)	0·4
**FINDRISC**	East Asian	246/756	0·105 (-0·025 - 0·234)	0·1	9·8 (-2·8 - 22·4)	0·1	0·6 (-2·3 - 3·6)	0·7
**FINDRISC**	Other	728/2,625	0·153 (0·068 - 0·238)	<0·001	14·8 (6·4 - 23·3)	<0·001	0·5 (-0·8 - 1·8)	0·4
**FINDRISC**	South Asian	840/1,553	0·138 (0·07 - 0·207)	<0·001	12·7 (6·1 - 19·3)	<0·001	1·1 (-0·7 - 3)	0·2
**FINDRISC**	Lifelines	8,947/23,739	-0·028 (-0·064 - 0·007)	0·1	-2·8 (-6·3 - 0·7)	0·1	0 (-0·5 - 0·4)	0·8
**AUSDRISK**	White	32,084/119,328	0·061 (0·045 - 0·076)	<0·001	5·9 (4·4 - 7·4)	<0·001	0·1 (-0·1 - 0·3)	0·2
**AUSDRISK**	Black	1,129/1,788	0·035 (-0·083 - 0·153)	0·6	3·4 (-8·2 - 15·1)	0·6	0 (-1·8 - 1·9)	1
**AUSDRISK**	Caribbean	191/1,066	0·061 (-0·037 - 0·158)	0·2	5·7 (-3·9 - 15·3)	0·2	0·3 (-1·5 - 2·2)	0·7
**AUSDRISK**	East Asian	236/527	0 (-0·169 - 0·169)	1	0 (-16·6 - 16·6)	1	0 (-3·2 - 3·2)	1
**AUSDRISK**	Other	600/2,235	0·264 (0·153 - 0·374)	<0·001	25·6 (14·7 - 36·6)	<0·001	0·7 (-0·8 - 2·2)	0·3
**AUSDRISK**	South Asian	585/1,418	0·082 (-0·006 - 0·171)	0·07	7·8 (-0·9 - 16·4)	0·08	0·5 (-1·5 - 2·4)	0·6
**AUSDRISK**	Lifelines	7,450/23,206	0·004 (-0·037 - 0·044)	0·9	0·4 (-3·7 - 4·4)	0·9	0 (-0·4 - 0·5)	1

Supplementary Table S10B. Reclassification analysis comparing our Questionnaire & Basic measurements models to FINDRISC and AUSDRISK. Reclassification events % correspond to our models' net percentage of reclassified individuals with T2D compared to the clinically established tools. Reclassification of events per 10,000 events corresponds to the net number of T2D cases reclassified when screening 10,000 cases. Positive reclassification events indicate that our models correctly reclassify more cases than the other two clinical tools, whereas negative events indicate the opposite. Reclassification percentages (%) are represented along with the 95% CI.

Risk model	Ethnicity	N high/low risk	NRI	NRI p-value	Reclassification events %	Reclassification events p-value	Reclassification non-events %	Reclassification non-events p-value
**FINDRISC**	White	37,718/145,351	0·108 (0·096 - 0·12)	<0·001	10·5 (9·3 - 11·6)	<0·001	0·3 (0·1 - 0·5)	<0·001
**FINDRISC**	Black	1,017/2,586	0 (-0·103 - 0·103)	1	0 (-10·2 - 10·2)	1	0 (-1·3 - 1·3)	1
**FINDRISC**	Caribbean	488/1,182	0·129 (0·035 - 0·223)	0·007	12·2 (3·1 - 21·4)	0·009	0·7 (-1·4 - 2·8)	0·5
**FINDRISC**	East Asian	281/752	0·115 (0·006 - 0·224)	0·04	10·8 (0·2 - 21·3)	0·05	0·7 (-1·9 - 3·4)	0·6
**FINDRISC**	Other	925/2,513	0·144 (0·063 - 0·225)	<0·001	13·9 (6 - 21·9)	<0·001	0·5 (-0·9 - 1·8)	0·5
**FINDRISC**	South Asian	851/1,562	0·076 (0·016 - 0·137)	0·01	7 (1·2 - 12·8)	0·02	0·6 (-1·1 - 2·4)	0·5
**FINDRISC**	Lifelines	11,772/20,914	0·041 (0·013 - 0·069)	0·004	4 (1·2 - 6·8)	0·005	0·1 (-0·3 - 0·5)	0·7
**AUSDRISK**	White	33,641/117,771	0·063 (0·049 - 0·078)	<0·001	6·2 (4·7 - 7·6)	<0·001	0·1 (0 - 0·3)	0·1
**AUSDRISK**	Black	797/2,120	0·174 (0·036 - 0·313)	0·01	17·2 (3·5 - 31)	0·01	0·2 (-1·4 - 1·8)	0·8
**AUSDRISK**	Caribbean	371/886	0·091 (0·001 - 0·18)	0·05	8·6 (-0·1 - 17·2)	0·05	0·5 (-1·8 - 2·9)	0·7
**AUSDRISK**	East Asian	275/488	0·103 (-0·015 - 0·221)	0·09	9·8 (-1·6 - 21·1)	0·09	0·6 (-2·7 - 3·8)	0·7
**AUSDRISK**	Other	702/2,133	0·25 (0·142 - 0·359)	<0·001	24·4 (13·6 - 35·1)	<0·001	0·7 (-0·9 - 2·3)	0·4
**AUSDRISK**	South Asian	611/1,392	0·064 (-0·022 - 0·15)	0·1	6 (-2·3 - 14·4)	0·2	0·4 (-1·5 - 2·3)	0·7
**AUSDRISK**	Lifelines	11,032/19,624	-0·019 (-0·049 - 0·011)	0·2	-1·9 (-4·9 - 1·1)	0·2	0 (-0·5 - 0·4)	0·9

Supplementary Table S10C. Reclassification analysis comparing our Questionnaire, Basic measurements, and Biomarkers models to FINDRISC and AUSDRISK. Reclassification events % correspond to our models' net percentage of reclassified individuals with T2D compared to the clinically established tools. Reclassification of events per 10,000 events corresponds to the net number of T2D cases reclassified when screening 10,000 cases. Positive reclassification events indicate that our models correctly reclassify more cases than the other two clinical tools, whereas negative events indicate the opposite. Reclassification percentages (%) are represented along with the 95% CI.

Risk model	Ethnicity	N high/low risk	NRI	NRI p-value	Reclassification events %	Reclassification events p-value	Reclassification non-events %	Reclassification non-events p-value
**FINDRISC**	White	29,517/149,308	0·235 (0·22 - 0·249)	<0·001	22·8 (21·4 - 24·2)	<0·001	0·7 (0·5 - 0·9)	<0·001
**FINDRISC**	Black	485/3,050	0·225 (0·075 - 0·375)	0·003	22·2 (7·3 - 37·2)	0·004	0·3 (-0·9 - 1·4)	0·6
**FINDRISC**	Caribbean	289/1,341	0·279 (0·175 - 0·384)	<0·001	26·4 (16·1 - 36·7)	<0·001	1·5 (-0·4 - 3·3)	0·1
**FINDRISC**	East Asian	154/819	0·284 (0·15 - 0·418)	<0·001	26·6 (13·4 - 39·7)	<0·001	1·9 (-0·7 - 4·4)	0·1
**FINDRISC**	Other	482/2,902	0·372 (0·277 - 0·468)	<0·001	36 (26·5 - 45·4)	<0·001	1·3 (-0·1 - 2·6)	0·07
**FINDRISC**	South Asian	569/1,738	0·334 (0·257 - 0·41)	<0·001	30·6 (23·2 - 38)	<0·001	2·8 (0·8 - 4·8)	0·007
**FINDRISC**	Lifelines	5,830/26,388	0·16 (0·117 - 0·204)	<0·001	15·8 (11·4 - 20·1)	<0·001	0·3 (-0·2 - 0·7)	0·2
**AUSDRISK**	White	22,427/125,440	0·209 (0·191 - 0·226)	<0·001	20·4 (18·6 - 22·2)	<0·001	0·5 (0·3 - 0·7)	<0·001
**AUSDRISK**	Black	739/2,112	0·105 (-0·043 - 0·252)	0·2	10·3 (-4·3 - 25)	0·2	0·1 (-1·5 - 1·7)	0·9
**AUSDRISK**	Caribbean	240/999	0·268 (0·161 - 0·375)	<0·001	25·4 (15 - 35·8)	<0·001	1·5 (-0·9 - 3·8)	0·2
**AUSDRISK**	East Asian	127/612	0·129 (-0·027 - 0·285)	0·1	12·2 (-3·2 - 27·6)	0·1	0·7 (-1·9 - 3·3)	0·6
**AUSDRISK**	Other	338/2,452	0·494 (0·382 - 0·607)	<0·001	48·1 (36·9 - 59·2)	<0·001	1·4 (-0·2 - 2·9)	0·08
**AUSDRISK**	South Asian	335/1,579	0·357 (0·253 - 0·462)	<0·001	33·6 (23·3 - 43·9)	<0·001	2·1 (0·1 - 4·1)	0·04
**AUSDRISK**	Lifelines	7,460/22,768	0·16 (0·118 - 0·201)	<0·001	15·7 (11·6 - 19·8)	<0·001	0·3 (-0·2 - 0·8)	0·3
